# FmvB: A *Francisella tularensis* Magnesium-Responsive Outer Membrane Protein that Plays a Role in Virulence

**DOI:** 10.1371/journal.pone.0160977

**Published:** 2016-08-11

**Authors:** Xiaojun Wu, Guoping Ren, William T. Gunning, David A. Weaver, Andrea L. Kalinoski, Sadik A. Khuder, Jason F. Huntley

**Affiliations:** 1 Department of Medical Microbiology and Immunology, University of Toledo College of Medicine and Life Sciences, Toledo, OH, United States of America; 2 Department of Pathology and Electron Microscopy Facility, University of Toledo College of Medicine and Life Sciences, Toledo, OH, United States of America; 3 Department of Surgery and Advanced Microscopy and Imaging Center, University of Toledo College of Medicine and Life Sciences, Toledo, OH, United States of America; 4 Department of Medicine, University of Toledo College of Medicine and Life Sciences, Toledo, OH, United States of America; Midwestern University, UNITED STATES

## Abstract

*Francisella tularensis* is the causative agent of the lethal disease tularemia. Despite decades of research, little is understood about why *F*. *tularensis* is so virulent. Bacterial outer membrane proteins (OMPs) are involved in various virulence processes, including protein secretion, host cell attachment, and intracellular survival. Many pathogenic bacteria require metals for intracellular survival and OMPs often play important roles in metal uptake. Previous studies identified three *F*. *tularensis* OMPs that play roles in iron acquisition. In this study, we examined two previously uncharacterized proteins, FTT0267 (named *fmvA*, for *F**rancisella* metal and virulence) and FTT0602c (*fmvB*), which are homologs of the previously studied *F*. *tularensis* iron acquisition genes and are predicted OMPs. To study the potential roles of FmvA and FmvB in metal acquisition and virulence, we first examined *fmvA* and f*mvB* expression following pulmonary infection of mice, finding that *fmvB* was upregulated up to 5-fold during *F*. *tularensis* infection of mice. Despite sequence homology to previously-characterized iron-acquisition genes, FmvA and FmvB do not appear to be involved iron uptake, as neither *fmvA* nor *fmvB* were upregulated in iron-limiting media and neither Δ*fmvA* nor Δ*fmvB* exhibited growth defects in iron limitation. However, when other metals were examined in this study, magnesium-limitation significantly induced *fmvB* expression, Δ*fmvB* was found to express significantly higher levels of lipopolysaccharide (LPS) in magnesium-limiting medium, and increased numbers of surface protrusions were observed on Δ*fmvB* in magnesium-limiting medium, compared to wild-type *F*. *tularensis* grown in magnesium-limiting medium. RNA sequencing analysis of Δ*fmvB* revealed the potential mechanism for increased LPS expression, as LPS synthesis genes *kdtA* and *wbtA* were significantly upregulated in Δ*fmvB*, compared with wild-type *F*. *tularensis*. To provide further evidence for the potential role of FmvB in magnesium uptake, we demonstrated that FmvB was outer membrane-localized. Finally, Δ*fmvB* was found to be attenuated in mice and cytokine analyses revealed that Δ*fmvB-*infected mice produced lower levels of pro-inflammatory cytokines, including GM-CSF, IL-3, and IL-10, compared with mice infected with wild-type *F*. *tularensis*. Taken together, although the function of FmvA remains unknown, FmvB appears to play a role in magnesium uptake and *F*. *tularensis* virulence. These results may provide new insights into the importance of magnesium for intracellular pathogens.

## Introduction

*Francisella tularensis* is an intracellular Gram-negative bacterium and the etiological agent of lethal disease tularemia. *F*. *tularensis* is a zoonotic pathogen that can be transmitted from infected animals to humans via multiple routes (*e*.*g*. skin contact, ingestion, inhalation of aerosols) and tick transmission has been well-documented [1]. *F*. *tularensis* has three subspecies: *F*. *tularensis* subsp. *tularensis*, *F*. *tularensis* subsp. *holarctica*, and *F*. *tularensis* subsp. *mediasiatica*. Of these, only subsp. *tularensis* and subsp. *holarctica* cause human disease [2]. Subspecies *tularensis*, also known as Type A, is the most virulent and is found exclusively in North America [1, 3]. Type A strains are extremely infectious, with fewer than 25 organisms capable of causing severe disease and death in humans [4]. *Francisella tularensis* subsp. *holarctica*, also known as Type B, causes similar clinical disease in humans but has a higher infectious dose (<10^3^ CFU) and lower mortality rates than Type A strains [3, 5]. *F*. *tularensis* has been classified as a Tier 1 Select Agent, indicating that it poses a severe threat to human and animal health and has the greatest risk of being developed into a bioweapon [1]. Although an attenuated Type B strain was developed and tested as a potential live vaccine strain (LVS), it is not FDA-approved due to safety and efficacy concerns [6]. Therefore, studies to identify *F*. *tularensis* virulence factors and develop safe and effective vaccines are urgently needed. Given that *F*. *tularensis* can infect a wide variety of cell types [7–10], *F*. *tularensis* also can serve as a model pathogen to help understand bacterial virulence and intracellular survival strategies for other pathogens.

Acquisition of metals is important for pathogenic bacteria to survive inside the host [11–13]. Cations are coenzymes for many bacterial metabolic processes, are required to maintain bacterial membrane integrity, and protect against damage from molecules such as reactive oxygen species (ROS) and nitric oxide (NO) [12, 14–16]. To protect against bacterial infections, the host is a metal-limiting environment [17, 18]. For example, host iron is primarily sequestered in transferrin (TF), lactoferrin (LF), ferritin, and heme [14, 19]. In addition, ferric (Fe^3+^) iron is the predominant and most stable form of iron, but its solubility is extremely low at pH 7 (10^−17^ M), while bacteria generally need 10^−5^ to 10^−7^ M Fe^3+^ to achieve optimal growth [18]. Many pathogenic bacteria possess multiple mechanisms to import metals from the host during infection. Bacterial metal transporters typically have low affinity and specificity and thus are constitutively expressed to import a variety of metals [12]. For example, the Trk K^+^ transporter in *E*. *coli* has low affinity and is constitutively expressed. Bacteria also express high-affinity metal transporters which are inducible and only activated during metal scarcity. The Kdp K^+^ transporter in *E*. *coli* is an inducible and high-affinity ATP-dependent transporter [20]. Finally, many pathogenic bacteria encode siderophores which facilitate uptake of iron inside the host, which has been demonstrated to be an iron-limiting environment [14, 18].

The *F*. *tularensis* genome contains 5 homologous genes, FTT0025c, FTT0267, FTT0602c, FTT0918, and FTT0919, that share 40–50% sequence identity [21]. Of these, FTT0025c (FslE) and FTT0918 (FupA) have been shown to be involved in iron acquisition [13, 22–26]. However, despite a high degree of homology between the genomes of *F*. *tularensis* Type A and Type B strains, their iron acquisition systems appear to function distinctly. For example, in the Type A strain SchuS4, FTT0025c (FslE) is required for siderophore-dependent ferric (Fe^3+^) iron uptake [25] and FTT0918 (FupA) is required for ferrous (Fe^2+^) iron uptake [23]. In contrast, in the Type B strain LVS, the FupAB protein (FTL0439; the result of a recombination event between *fupA* and *fupB* that has been correlated with LVS attenuation [27]) is required for siderophore-dependent Fe^3+^ and siderophore-independent Fe^2+^ uptake [26, 28]. Studies in macrophages and mice have demonstrated that SchuS4 FTT0918 (FupA) and LVS FTL0439 (FupAB) play important roles in virulence [23, 24, 26], but deletion of SchuS4 FTT0919 (FupB) does not seem to affect iron acquisition or virulence [22, 23]. Despite these previous studies, no work has been performed to characterize the roles of FTT0267 and FTT0602 in iron acquisition or virulence.

Based on sequence homology to FslE, FupA, and FupB, we hypothesized that FTT0267 (designated herein as FmvA, for *F**rancisella* metal and virulence gene A) and FTT0602 (designated herein as FmvB) may be involved in *F*. *tularensis* metal uptake and virulence. In this study, we demonstrated that both *fmvA* and *fmvB* were upregulated during *F*. *tularensis* SchuS4 infections of mice, indicating that these genes may play important roles in *F*. *tularensis* virulence. However, neither *fmvA* nor *fmvB* were upregulated in iron-limiting medium and Δ*fmvA* and Δ*fmvB* did not exhibit growth defects when grown in iron-limiting medium, indicating that FmvA and FmvB do not play roles in iron acquisition. Interestingly, careful examination of *F*. *tularensis* growth in the absence of other cations revealed that *fmvB* was significantly upregulated in magnesium limitation, suggesting that FmvB might be involved in magnesium uptake. Further, bioinformatic prediction programs indicated that FmvB likely forms a beta barrel in the outer membrane and *F*. *tularensis* membrane localization studies confirmed that FmvB is outer membrane-localized. Because of the known importance of magnesium to neutralize LPS negative charges in the outer membrane, we next examined LPS expression in *ΔfmvB*, finding that *ΔfmvB* expressed 50% more LPS than wild-type *F*. *tularensis*. Finally, we found that Δ*fmvB* was significantly attenuated in a mouse pulmonary infection model. Detailed analysis of Δ*fmvB*-infected mice revealed reduced pro-inflammatory cytokine expression and reduced bacterial burdens on day 5 post-infection, which correlated with the observed attenuation of Δ*fmvB*. In summary, although FmvA and FmvB do not appear to be involved in iron uptake, FmvB is an outer membrane-localized protein that is upregulated in magnesium limitation, indicating its potential role in magnesium uptake, and FmvB is required for the full virulence of *F*. *tularensis*.

## Materials and Methods

### Bacterial strains and culture conditions

*F*. *tularensis* culture conditions have been previously described [29]. Following all federal and institutional select agent and biosafety regulations, *F*. *tularensis* Type A strain SchuS4 and *F*. *tularensis* Type B strain LVS were obtained from BEI Resources. All experiments with SchuS4 were performed under strict BSL3 containment conditions at the University of Toledo Health Science Campus BSL3 laboratory, including the use of liquid-impervious personal protective equipment (PPE) and powered air purifying respirators (PAPRs). All experiments with LVS were performed under BSL2 containment conditions. All *F*. *tularensis* stock (including both LVS and SchuS4) cultures first were grown at 37°C with 5% CO_2_ on supplemented Mueller-Hinton agar (sMHA): Mueller-Hinton broth (Becton Dickinson) was mixed with 1% (wt/vol) tryptone, 0.5% (wt/vol) sodium chloride, and 1.6% (wt/vol) Bacto Agar (Becton Dickinson), autoclaved, cooled to 50°C, and further supplemented with 0.1% (wt/vol) glucose, 0.025% (wt/vol) iron pyrophosphate, 2.5% (vol/vol) donor calf serum (Mediatech), and 2% (vol/vol) IsoVitaleX (Becton Dickinson). Following 16 to 24 h of growth on sMHA, individual *F*. *tularensis* colonies were inoculated into supplemented Mueller-Hinton broth (sMHB): Mueller-Hinton broth was mixed with 182 mg/L calcium chloride dihydrate, and 210 mg/L magnesium chloride hexahydrate, autoclave sterilized, and further supplemented with 0.1% (wt/vol) glucose, 0.025% (wt/vol) iron pyrophosphate, and 2% (vol/vol) IsoVitaleX before use. *E*. *coli* S17-1 was used as the donor strain for *F*. *tularensis* isogenic gene deletion construct conjugation. S17-1 bacteria were grown in LB broth or on LB agar at 37°C, supplemented as needed with 30 mg/L kanamycin (kan).

### Sequence comparisons and bioinformatics predictions

Amino acid sequence comparisons of FslE (FTT_0025c), FupA (FTT_0918), FupB (FTT_0919), FmvA (FTT_0267), and FmvB (FTT_0602c) were performed using BLASTP analysis (http://blast.ncbi.nlm.nih.gov) and ClustalW alignment (MacVector version 12.6.0). Bacterial protein localization was predicted by PSORTb version 3.0.2 (http://www.psort.org/psortb/). Beta-barrel structural predictions were performed using PROFtmb (http://cubic.bioc.columbia.edu/services/proftmb/), BOMP (http://www.bioinfo.no/tools/bomp), TMBB-DB (http://beta-barrel.tulane.edu/), and TMB-Hunt (http://www.bioinformatics.leeds.ac.uk/~andy/betaBarrel/AACompPred/aaTMB_Hunt.cgi). Two-dimensional topology models of FmvA and FmvB were generated by PRED-TMBB (http://biophysics.biol.uoa.gr/PRED-TMBB/) and viewed using TMRPres2D (http://biophysics.biol.uoa.gr/TMRPres2D/). Three-dimensional beta barrel models of FmvA and FmvB were generated by TMBpro (http://tmbpro.ics.uci.edu/) and viewed using Swiss-PdbViewer (http://spdbv.vital-it.ch/).

### Mouse infections

All animal studies were approved by the University of Toledo Institutional Animal Care and Use Committee (IACUC). Mouse infections were performed as previously described with minor modifications [30]. *F*. *tularensis* was grown on Brain Heart Infusion (BHI) agar for 20 to 24 h, suspended in PBS, and diluted to either 30 CFU/20 μl for SchuS4 studies or from 5 X 10^3^ to 1 X 10^4^ CFU/20 μl for LVS studies based on OD_600_ measurements and previous bacterial enumeration studies. Groups of four to eight C3H/HeN female mice (6–8 weeks old; National Cancer Institute) were anaesthetized with a ketamine-xylazine sedative (0.6 mg ketamine and 0.06 mg xylazine per mouse) and intranasally (i.n.) inoculated with 20 μl of either wild-type or isogenic mutant *F*. *tularensis* strains. Inocula were serially diluted and plated in quadruplet on sMHA plates to confirm CFU. For *F*. *tularensis* gene expression studies in mice, four mice per group were euthanized on day 1 through day 5 post-infection, lungs, livers, and spleens were aseptically harvested and individually transferred to sterile Whirl-pack bags (Nasco), snap frozen in liquid nitrogen, organs were homogenized, suspended in TRIzol (Invitrogen), and RNA was purified following the manufacturer’s instructions. For survival studies, mice were monitored at least twice daily (once in the morning and once in the late afternoon) for signs of morbidity and mortality, with health status scores (numerical scale from 1 to 5 with 1 indicating healthy mice and 5 indicating mice found dead) being recorded for each experimental group. Any mouse observed at health status 4 (mice extremely sick or moribund with ruffled fur, conjunctivitis, labored breathing, and unable to move when gently prodded) was immediately euthanized by an intraperitoneal injection of a ketamine-xylazine sedative (1 mg ketamine and 0.1 mg xylazine per mouse) followed by cervical dislocation. Because of the acute and lethal nature of *F*. *tularensis* infections, there were occasions when mice were observed at health status 3 (mice appeared sick with ruffled fur, conjunctivitis, and slowed movement but still mobile) during a late afternoon health check but were found dead (health status 5) the next morning. To quantitate differences in bacterial tissue burdens for *F*. *tularensis* wild-type- and isogenic mutant-infected mice, 4 mice per group were euthanized on days 2 and 5 post-infection, lungs, livers, and spleens were aseptically harvested, individually transferred to sterile Whirl-pack bags, weighed, 25 μl of PBS per mg of tissue was added to each bag, tissues were homogenized, 10-fold serially-diluted in PBS, and 100 μl of each dilution was plated in duplicate onto sMHA plates. Following 48 to 72 h of incubation, the number of CFU per plate was determined for each dilution, and the average number of CFU/mg of tissue was calculated based on the CFU count from the plates and original tissue weights. For multiplex cytokine quantitation from non-infected and *F*. *tularensis*-infected mice, lungs, livers, and spleens were harvested from either non-infected (age-matched) or infected mice on days 2 and 5 post-infection, individually transferred to sterile Whirl-pack bags, suspended in 25 μl of ice-cold lysis buffer (150 mM NaCl, 5 mM EDTA, 10 mM Tris base, and 1 ml of protease inhibitor cocktail III [A.G. Scientific] per 200 ml lysis buffer) per mg of tissue, homogenized, centrifuged at 4,800 X *g* for 10 min at 4°C to remove large particulates, filter sterilized using 0.22 μm syringe filters (EMD Millipore), and stored at -80°C until use.

### RNA purification and quantitative RT-PCR (qRT-PCR)

Total bacterial RNA was extracted from either *F*. *tularensis* (SchuS4 or LVS)-infected mouse tissues or *F*. *tularensis* (SchuS4 or LVS) grown in sMHB or metal-limiting medium using TRIzol (Invitrogen) following the manufacturer’s instructions. Purified RNA was treated with DNase I (Ambion) to remove genomic DNA contamination. After DNA digestion, RNA was further purified using the RNeasy RNA Mini Kit (Qiagen). For RNA purified from *F*. *tularensis*-infected mouse tissues, MICROBEnrich (Ambion) was used to remove mammalian RNA following the manufacturer’s instructions. SuperScript Vilo (Life Technologies) was used to reverse transcribe 2 μg of RNA from each RNA sample. Quantitative real-time PCR (qRT-PCR) reactions contained cDNA (either a 25-fold dilution of cDNA from SchuS4-infected mouse tissues or a 32-fold dilution of cDNA from metal limiting media), 0.2 units of HotStarTaq Plus DNA Polymerase (Qiagen), 0.4X of 10,000X SYBR Green (Life Technologies), and 1X qRT-PCR buffer (including 1X Enzyme Diluent [Idaho Technology], 1X of Buffer 10X w/BSA-30 mM MgCl_2_ [Idaho Technology], 10 mM dNTP Mix [Thermo Scientific], molecular grade water [Corning], and 0.1 μM of each primer). qRT-PCR primers were designed using PrimerQuest (Integrated DNA Technologies). Primers used for assessing gene expression in *F*. *tularensis* SchuS4-infected mouse tissues and from *F*. *tularensis* LVS in metal limiting media are listed in Table A in S1 File. DNA gyrase subunit α (*gyrA*, FTT1575c and FTL0533) generally served as the internal control. However, because of inherent differences between SchuS4 and LVS gene expression *in vitro* and *in vivo*, two different ‘housekeeping’ genes were tested to verify qRT-PCR analysis. For analysis of SchuS4 gene expression changes in infected mice, DNA gyrase subunit α (*gyrA*; FTT1575c) and RNA polymerase α subunit (*rpoA*; FTT0350) were used as internal controls. Similar trends were observed when using either ‘housekeeping’ gene. For analysis of LVS gene expression changes in metal-limiting media, DNA gyrase subunit α (*gyrA*; FTL0533) and DNA helicase II (*uvrD*, FTL1656) were used as internal controls. Similar tends were observed when using either ‘housekeeping’ gene. All qRT-PCR reactions were performed in triplicate, three independent experiments were analyzed by qRT-PCR, and non-reverse transcribed RNA samples served as negative controls to assess genomic DNA contamination. qRT-PCR reactions were performed using an Applied Biosystems 7500 real-time PCR system and data were analyzed using Applied Biosystems 7500 software v2.0.6. Relative numbers of *fslE*, *fupA*, *fupB*, *fmvA*, and *fmvB* mRNA transcripts during SchuS4 infection of mice were calculated based on *gyrA* mRNA transcripts and are presented as fold change relative to their expression in sMHB laboratory medium. Relative numbers of *fslE*, *fupAB*, *fmvA*, and *fmvB* mRNA transcripts in metal-limiting media were calculated based on *gyrA* mRNA transcripts and are presented as fold change relative to their expression in metal replete medium.

### *F*. *tularensis* isogenic deletions (knockouts) generation

A detailed procedure to generate isogenic mutants by homologous recombination in *F*. *tularensis* was previously described [31]. Briefly, 500 to 1000 bp upstream and downstream regions flanking the gene of interest were amplified from *F*. *tularensis* LVS genomic DNA with engineered ApaI restriction sites on the 5’ end of each amplicon and engineered sequences homologous to the FLP recombination target (FRT)-flanked Pfn-kanamycin resistance cassette (FRT-Pfn-*kan*-FRT) on the 3’ end of each amplicon. Next, FRT-Pfn-*kan*-FRT was PCR amplified from plasmid pLG66a to replace the gene of interest [32]. Splicing-overlap extension (SOE) PCR was used to fuse together the three PCR amplicons in the following order: upstream flanking region, FRT-Pfn-*kan*-FRT, downstream flanking region. The resulting amplicon and suicide plasmid pTP163 [33] were digested with *Apa*I (New England Biolabs) and then ligated together using T4 DNA ligase (New England Biolabs). The resulting gene deletion (knockout) constructs were sequence-verified to ensure that no mutations had been introduced into either the upstream or downstream flanking regions. Knockout constructs were transformed into *E*. *coli* S17.1 and conjugation was performed with *F*. *tularensis* LVS on sMHA plates without antibiotics. The transconjugants initially were recovered on modified chocolate agar (CHOC) supplemented with 200 mg/L hygromycin (hyg) and 100 mg/L polymyxin B (pxb). CHOC was prepared by mixing Mueller-Hinton medium with 1% (wt/vol) tryptone, 0.5% (wt/vol) NaCl, and 1.6% (wt/vol) agar. After autoclave sterilization, the solution was cooled to 50°C and further supplemented with 1% (wt/vol) bovine hemoglobin (Neogen) and 1% (vol/vol) IsoVitaleX. Colonies from CHOC-hyg-pxb plates were then passaged on sMHA supplemented with 10 mg/L kanamycin (kan) to select for colonies that integrated the pTP163 suicide plasmid (including the kan resistance cassette) and subsequently passaged on sMHA supplemented with 10 mg/L kan and 8% (wt/vol) sucrose to select for clones that released the knockout construct containing both the target gene and the *sacB* sucrose sensitivity marker. Finally, sucrose- and kan- resistant colonies were replica plated onto sMHA containing either 200 mg/L hyg or 10 mg/L kan to confirm kan resistance (loss of gene of interest) and hyg sensitivity (loss of pTP163 suicide plasmid). Diagnostic PCR was performed on kan-resistant colonies to verify the presence of FRT-Pfn-*kan*-FRT and the absence of the gene of interest. To generate double gene deletion mutants in *F*. *tularensis*, FRT-Pfn-kan-FRT was removed from single gene deletion mutants by electroporating the shuttle plasmid pTP405 (generous gift from Drs. Gregory Robertson and Michael Norgard, University of Texas Southwestern Medical Center; [33]) which encodes the Flp recombinase that recognizes and cleaves FRT-Pfn-*kan*-FRT from the genome. pTP405 was electroporated into single gene deletion mutants and bacteria were incubated on sMHA containing 200 mg/L hyg to select for transformants. Hyg-resistant colonies were passaged 2 to 3 times on sMHA without antibiotics, then patched on sMHA without antibiotics or sMHA containing either 200 mg/L hyg or 10 mg/L kan plates to confirm sensitivity to both antibiotics. The resulting hyg- and kan-sensitive colonies were then used to generate a second deletion mutant following the procedure outlined above.

### *fmvB* complementation *in-trans*

To aid in FmvB subcellular localization in *F*. *tularensis* LVS, a C-terminal 6x histidine-tagged FmvB was re-introduced into Δ*fmvB*. Full length *fmvB* was PCR-amplified from LVS genomic DNA using primers 5’-c*fmvB*-pQE-60: 5’-GCGCCCATGGATGAAGTATATTTACAAAAAATTATTAATATATAGTTTTTTTATGTG-3’ and 3’-c*fmvB*-pQE-60: 5’- GCGCGGATCCCATCATTATAAAATTTTTGATATGCATATTCAGG-3’. The resulting amplicon was double-digested with NcoI and BamHI restriction enzymes (New England Biolabs) and ligated into similarly-digested pQE-60 (Qiagen) using T4 DNA Ligase (New England Biolabs). The ligation product was transformed into 10-β *E*. *coli* (New England Biolabs) and positive transformants were selected on LB agar supplemented with 100 mg/L ampicillin (amp). Plasmids were purified from Amp-resistant colonies using QIAprep spin Miniprep kit (Qiagen), diagnostic digestion was performed to confirm the presence of correctly-sized inserts, and DNA sequencing was performed to confirm the *fmvB* sequence. The *fmvB* coding sequence and in-frame C-terminal 6x histidine tag were PCR-amplified from pQE-60 using primers 5’-c*fmB*-pFNLTP6: 5’- GCGCCTCGAGATGAAGTATATTTACAAAAAATTATTAATATATAGTTTTTTTATGTG-3’ and 3’-c*fmB*-pFNLTP6: 5’- GCGCGGATCCTTAGTGATGGTGATGGTGATG. The resulting amplicon was double digested with XhoI and BamHI restriction enzymes (New England Biolabs) and ligated into similarly digested pFNLTP6-gro-GFP [34] (GFP removed by XhoI and BamHI digestion) using T4 DNA Ligase (New England Biolabs). The ligation product, pFNLTP6-gro-*fmvB*-6xHis, was transformed into NEB 10-β *E*. *coli* (New England Biolabs) and positive transformants were selected on LB agar supplemented with 50 mg/L kan. Plasmids were purified from kan-resistant colonies using QIAprep spin Miniprep kit (Qiagen), diagnostic digestion was performed to confirm the presence of correctly-sized inserts, and DNA sequencing was performed to confirm the *fmvB-*6x histidine tag fusion sequence. pFNLTP6-gro-*fmvB*-6xHis was transformed into Δ*fmvB* by electroporation and transformants were selected on sMHA containing 10 mg/L kan. Δ*fmvB* that was complemented with pFNLTP6-gro-*fmvB*-6xHis is designated hereafter as Δ*fmvB* + *fmvB*-6xHis.

### Bacterial growth in metal-limiting media

For recovery of wild-type *F*. *tularensis* LVS and isogenic mutants from -80°C storage, bacterial strains were grown in chemically defined liquid or agar media (CDM), as previously described [35]. For all metal depletion studies, bacteria were initially grown on CDM agar, then inoculated into liquid CDM that had been treated for 12 h with 10 g/L Chelex 100 resin (low chelex-treated; Bio-Rad), chelex resin was removed by a 0.22 μM filter, and the resulting low chelex-treated CDM was supplemented with 5 μM calcium chloride, 0.55 mM magnesium sulfate, and 2.5 μg/ml iron pyrophosphate (supp. low chelex-treated CDM). Following overnight growth in supp. low chelex-treated CDM, LVS and isogenic mutants were adjusted to an OD_600_ of 0.5, diluted 1:50 into low chelex-treated CDM, and grown for 16 h. Following 16 h of growth, cultures were washed three times with liquid CDM that had been treated for 4 h with 50 g/L Chelex 100 resin and chelex resin was removed by a 0.22 μM filter (high chelex-treated). The resulting bacteria were standardized to an OD_600_ of 0.5 and then diluted 1:50 into either high chelex-treated liquid CDM, supplemented with 5 μM calcium chloride, 0.55 mM magnesium sulfate, and 2.5 μg/ml iron pyrophosphate (CDM replete) or specific metal-depleted CDM media were prepared as follows: high chelex-treated CDM was supplemented with 0.55 mM magnesium sulfate, 5 μM calcium chloride, and 0.75 μg/ml iron pyrophosphate (low iron; -Fe;); high chelex-treated CDM was supplemented with 0.55 mM magnesium sulfate and 2.5 μg/ml iron pyrophosphate (no calcium; -Ca); high chelex-treated CDM was supplemented with 0.55 mM magnesium sulfate and 0.75 μg/ml iron pyrophosphate (no calcium and low iron; -Ca, -Fe); high chelex-treated CDM was supplemented with 2.5 μg/ml iron pyrophosphate and 5 μM calcium chloride (no magnesium; -Mg); high chelex-treated CDM was supplemented with 2.5 μg/ml iron pyrophosphate only (no magnesium or calcium; -Mg, -Ca); and high chelex-treated CDM was supplemented with 5 μM calcium chloride and 0.75 μg/ml iron pyrophosphate (no magnesium and low iron; -Mg, -Fe). For qRT-PCR analysis of *fslE*, *fupAB*, *fmvA*, and *fmvB* gene expression in various metal-depleted media, wild-type *F*. *tularensis* LVS was first cultured in low chelex-treated CDM for 16 h then cultured in high chelex-treated CDM with or without the indicated metals for 24 h before bacterial harvest and RNA purification.

To evaluate growth of Δ*fmvA*, Δ*fmvB*, Δ*fslE*, Δ*fupAB*, Δ*fmvB/*Δ*fslE*, and Δ*fmvB*/Δ*fupAB* in magnesium-limiting liquid medium, bacterial strains were cultured in supp. low chelex-treated CDM for 16 h, washed three times with high chelex-treated CDM, and inoculated into high chelex-treated CDM supplemented with 2.5 μg/ml iron pyrophosphate, 5 μM calcium chloride, and 27 μM magnesium sulfate (low magnesium; -Mg). Bacterial growth was monitored over a period of 50 h based on OD_600_ measurements. For examination of LPS expression in magnesium-limiting medium, wild-type LVS and Δ*fmvB* bacterial strains were grown in either replete CDM or high chelex-treated CDM supplemented with 2.5 μg/ml iron pyrophosphate, 5 μM calcium chloride, and 27 μM magnesium sulfate (low magnesium; -Mg) for 24 h before harvesting for immunoblot and flow cytometry analysis. For examination of bacterial morphology in magnesium-limiting medium, wild-type LVS and Δ*fmvB* bacterial strains were grown in high chelex-treated CDM supplemented with 2.5 μg/ml iron pyrophosphate, 5 μM calcium chloride, and 27 μM magnesium sulfate (low magnesium; -Mg) for 24 h before harvesting for electron microscopy visualization.

To evaluate growth of Δ*fmvA*, Δ*fmvB*, Δ*fupAB*, Δ*fmvA/*Δ*fupAB*, and Δ*fmvB*/Δ*fupAB* in iron-limiting liquid medium, bacterial strains were grown for 16 h in supp. low chelex-treated CDM, washed three times with low chelex-treated CDM without metal supplement, and then the bacteria were inoculated in low chelex-treated CDM supplemented with 5 μM calcium chloride, 0.55 mM magnesium sulfate, and 0.05 μg/ml iron pyrophosphate. Bacterial growth was monitored over a period of 50 h based on OD_600_ measurements. To evaluate growth of wild-type LVS, Δ*fmvA*, Δ*fmvB*, Δ*fupAB*, Δ*fmvA/*Δ*fupAB*, and Δ*fmvB*/Δ*fupAB* on iron-limiting agar, bacterial strains were inoculated onto either sMHA agar plates (iron replete) or iron-limiting CDM agar plates (CDM supplemented with 1% Bacto Agar and no additional iron). Bacterial strains first were cultured on iron replete CDM agar plates for 24 h, suspended in liquid CDM without iron pyrophosphate supplementation, OD_600_ of each bacterial strain was adjusted to 1.0, bacteria were serially-diluted 1:5 in liquid CDM without iron pyrophosphate supplementation, and 5 μl of each dilution was spotted onto either sMHA (iron replete) or iron-limiting CDM agar plates (CDM without iron pyrophosphate). Plates were incubated at 37°C with 5% CO_2_ for 72h before bacterial growth was examined.

### Antimicrobial sensitivity testing

Wild-type LVS and Δ*fmvB* were grown in the presence of gentamicin (Gibco), ciprofloxacin (Oxoid), tetracycline (Fisher BioReagents), SDS (Fisher BioReagents), Triton X-100 (Acros Organics), CTAB (MP Biomedicals), and ethidium bromide (Thermo Scientific) to examine potential membrane integrity defects for Δ*fmvB*. LVS and Δ*fmvB* were grown on sMHA plates overnight, suspended in PBS, the OD_600_ was adjusted to 0.2, and bacterial suspensions were evenly plated onto the entire surface of BHI plates using sterile cotton-tipped applicators. An autoclaved blotting paper disc (0.8 mm thick, 6.5 mm diameter; Whatman) was placed in the center of each inoculated BHI plate and the following compounds were added to separate discs: 5 μg/disc of gentamicin, ciprofloxacin, tetracycline, and ethidium bromide; 750 μg/disc of SDS and Triton X-100; and 50 μg/disc of CTAB. Plates were incubated at 37°C with 5% CO_2_ for 48 h before zone of inhibition measurements were measured. The average zones of inhibition (diameter in mm; including filter disk) to each compound were calculated for each bacterial strain. Next, wild-type LVS and Δ*fmvB* were incubated in the presence of the antimicrobial peptides hBD-3 (Anaspec), HNP-2 (Anaspec), polymyxin B (MP Biomedicals), and LL-37 (Anaspec) to examine potential membrane integrity defects for Δ*fmvB*. LVS and Δ*fmvB* first were grown on sMHA plates overnight, suspended in 10 mM sodium phosphate (pH 7.0) and 5% tryptic soy broth supplemented with 1% cysteine (TSB/C). Bacteria were adjusted to 10^6^ CFU/ml (based on OD_600_ measurements), and bacterial strains were incubated with each antimicrobial peptide and incubated at 37°C for 2 h. hBD-3, HNP-2, and polymyxin B were tested at 100 μg/ml and LL-37 was tested at 10 μg/ml. Following the 2 h incubation, each bacterial strain/antimicrobial peptide reaction was serially-diluted and plated in duplicate onto sMHA plates. Bacterial colonies were enumerated after 72 h of growth.

### Spheroplasting, osmotic lysis, and sucrose density gradient centrifugation

Spheroplasting, osmotic lysis, and sucrose density gradient centrifugation were performed as previously described [36] to determine the bacterial subcellular location of FmvB. Briefly, Δ*fmvB* and Δ*fmvB* (pFNLTP6-gro-*fmvB*-6xHis) complemented strain cultures were grown overnight in 1 L batches in sMHB to an OD_600_ of 0.3 to 0.4. Bacterial cultures were centrifuged at 7,500 X *g* for 30 min at 10°C to pellet the bacteria, supernatants were removed, and centrifuge bottles were briefly tapped on absorbent material to remove excess growth medium. To generate spheroplasts, bacterial pellets were sequentially suspended in 0.75 M sucrose (in 5 mM Tris, pH 7.5) with gentle mixing, 10 mM EDTA (in 5 mM Tris, pH 7.8) was then slowly added over the course of 10 min, bacteria were incubated with gentle stirring for 30 min at room temperature, lysozyme was slowly added over the course of 1 min to a final concentration of 200 μg/ml, and the bacteria were incubated for 30 min at room temperature with gently stirring. To osmotically lyse the bacteria, the above suspension was slowly diluted into a 4.5-fold excess volume of molecular-grade distilled water (Corning) over the course of 11 min and incubated for 30 min at room temperature with gentle stirring. The lysis solution was centrifuged at 7,500 X *g* for 30 min at 10°C to pellet intact cells and debris. Supernatants were collected and centrifuged at 182,500 X *g* (37,000 rpm in F37L 8x100 Fiberlite Ultracentrifuge rotor, Thermo Scientific) for 2 h and 10 min at 4°C. Following centrifugation, supernatants were removed, tubes were briefly tapped on absorbent material to remove excess supernatants, and total membrane pellets were gently resuspended in 7 to 8 ml of resuspension buffer (25% [wt/wt] sucrose, 5 mM Tris, 30 mM MgCl_2_, 1 tablet of Complete Mini EDTA-free protease inhibitor cocktail [Pierce], and 750 U Benzonase [Novagen]). Total membranes were suspended by gently inverting the solution for 30 min at room temperature. The DC protein assay (Bio-Rad) was performed to quantify protein concentration in membrane suspensions. Linear sucrose gradients were prepared by layering 1.8 ml of the following sucrose solutions (wt/wt; prepared in 5 mM EDTA, pH 7.5) into 14- by 95-mm ultracentrifuge tubes (Beckman) in the following order: 55%, 50%, 45%, 40%, 35%, and 30%. Total membrane suspensions were layered on top of each sucrose gradient, with less than 1.5 mg of protein loaded per gradient. Sucrose gradients were centrifuged in an SW40 swing bucket rotor at 256,000 X *g* (38,000 rpm) for 17 h minimum at 4°C. Upon completion of centrifugation, sucrose gradient tubes were removed from the rotor and 500 μl fractions immediately were collected from the bottom of each gradient by puncturing the bottom of each tube. The refractive index of each sucrose fraction was measured using a refractometer (Thermo Scientific) and specific density was correlated to g/ml [37]. Equal amounts of representative sucrose gradient fractions (1.18 g/ml for outer membrane vesicle fractions; 1.13 g/ml for inner membrane vesicle fractions) were examined by immunoblot analysis using FopA antiserum, SecY antiserum, or Penta-His HRP conjugate antibody (Qiagen) as described below.

### Immunoblotting

For detection of FopA (outer membrane control), SecY (inner membrane control), and 6x-histidine fusion tagged FmvB in sucrose gradient fractions, samples were diluted in SDS-PAGE loading buffer and boiled for 10 min. For comparison, whole cell lysates of Δ*fmvB* and Δ*fmvB* (pFNLTP6-gro-*fmvB*-6xHis) were prepared by suspension in SDS-PAGE loading buffer and boiling for 10 min. Molecular mass standards (Precision Plus protein all blue prestained protein standards; BioRad Laboratories) and protein samples were separated by SDS-PAGE, transferred to nitrocellulose, blots were incubated overnight in blot block (0.1% (vol/vol) Tween 20 and 2% (wt/vol) bovine serum albumin in PBS) at 4°C, and immunoblotting was performed using rat polyclonal antiserum specific for either *F*. *tularensis* FopA or *F*. *tularensis* SecY [36] or the Penta-His HRP conjugate antibody (Qiagen). FopA antiserum was diluted 1:10,000 in blot block, SecY antiserum was diluted 1:5000 in blot block, and Penta-His HRP conjugate antibody was diluted 1:10,000 in block. Blots were incubated with primary antisera for 2 h at room temperature, washed four times for 5 min each with blot washing buffer (0.05% [vol/vol] Tween 20 in PBS), FopA and SecY blots were incubated with goat anti-rat IgG (H+L)-HRP secondary antibody (Jackson ImmunoResearch Laboratories) for 1 h at room temperature, washed 3 times for 5 min each with blot washing buffer, washed one time for 5 min in PBS, and blots were developed by either colorimetric detection reagent (FopA; 0.06% [wt/vol] 4-chloro-1-naphthol (Sigma), 10.4% [vol/vol] methanol, and 0.13% [vol/vol] hydrogen peroxide in 200 mM NaCl and 50 mM Tris base) or chemiluminescent detection reagent (SecY and Penta-His HRP; SuperSignal Chemiluminescent Substrate; Thermo Scientific).

To compare LPS expression levels between LVS and Δ*fmvB* grown in magnesium-limiting medium, bacteria were harvested by centrifugation, adjusted to OD_600_ 0.8, suspended in SDS-PAGE loading buffer, and boiled for 10 min. Equal quantities of each protein sample were separated by SDS-PAGE, transferred to nitrocellulose, blots were incubated blocked overnight in blot block at 4°C, and immunoblotting was performed using either anti-*Francisella tularensis* LPS antibody FB11 (Abcam) or FopA antiserum (loading control). LPS antibody was diluted at 1:100,000 in blot block and FopA antibody was diluted at 1:10,000 in blot block. Blots were incubated with primary antibodies for 2 h at room temperature and washed four times for 5 min each with blot washing buffer. LPS blots were incubated for 1 h at room temperature (RT) with a 1:10,000 dilution of secondary rabbit anti-mouse immunoglobulin G (IgG)–horseradish peroxidase (HRP) antibody (Santa Cruz) and FopA blots were incubated with secondary antibody as described above. Blots were washed three times for 5 min each with blot washing buffer, washed one time for 5 min in PBS, and then developed by either colorimetric detection reagent (FopA) or chemiluminescent detection reagent (LPS) as described above. LPS expression levels were quantitated and compared based on densitometry analysis (ImageJ; https://imagej.nih.gov/ij/) of all reactive bands and were standardized based on FopA expression.

### Flow cytometry

The use of flow cytometry to quantitate LVS and Δ*fmvB* LPS surface expression was modified based on previously published studies in other bacteria [38, 39]. Briefly, LVS and Δ*fmvB* were cultured in magnesium-limiting medium for 24 h as described above, approximately 10^9^ bacteria were harvested by centrifugation at 6,000 ☓ *g* for 5 min, suspended in 1 ml PBS containing 5% (wt/vol) BSA, pelleted by centrifugation at 6,000 ☓ *g* for 5 min, and suspended in 500 μl of PBS containing 5% BSA and a 1:1,000 dilution of anti-*Francisella tularensis* LPS antibody FB11. Following 1 h of incubation at 4°C, bacteria were pelleted and washed three times with 1 ml of PBS containing 5% BSA and bacteria were suspended in 500 μl of PBS containing 5% BSA and a 1:500 dilution of Alexa Fluor 488 goat anti mouse IgG (H+L) secondary antibody. Following 1 h of incubation at 4°C, bacteria were pelleted and washed three times with 1 ml PBS containing 5% BSA, suspended in 1% (wt/vol) paraformaldehyde, incubated for 30 min, bacteria were pelleted and washed two times with PBS, and suspended in 500 μl of PBS. Bacteria immediately were analyzed by flow cytometry using FACSAria (BD Biosciences) with FACSDiva software (BD Biosciences) and data was analyzed using FlowJo (Tree Star).

### Electron microscopy

LVS and Δ*fmvB* for electron microscopy examination were cultured in magnesium-limiting medium as described above. Briefly, following overnight growth in magnesium limitation, approximately 10^8^ CFU of LVS or Δ*fmvB* were pelleted by centrifugation, washed twice with PBS, bacteria were fixed in 3% (vol/vol) glutaraldehyde (Electron Microscopy Sciences [EMS]) at room temperature for 2 h, and embedded in melted agarose. Bacterial samples were washed three times for 10 min each with fresh cacodylate buffer (pH 7.4; EMS). Samples were then immersed in 1% (wt/vol) osmium tetroxide (EMS) in *s*-collidine buffer (pH 7.4; EMS) at 4°C for 2 h at room temperature followed by three washes with fresh *s*-collidine buffer for 10 min each at room temperature. Tertiary fixation was employed using an aqueous saturated solution of uranyl acetate (pH 3.3; EMS) for 1 h at room temperature. Samples were dehydrated at room temperature using a graded series of ethanol washes: two washes with 30% ethanol for 10 min each; two washes with 50% ethanol for 10 min each; two washes with 70% ethanol for 10 min each; two washes with 90% ethanol for 10 min each; two washes with 100% ethanol for 10 min each; three washes with 100% acetone for 10 min each. Samples were then infiltrated with 50% acetone and 50% embedding media (Hard Plus Resin 812, EMS) for 8 h to overnight at room temperature. Samples were embedded in 100% embedding media (EMS) and allowed to polymerize for 8 h to overnight at 85°C, then sectioned at 85–90 nm, and visualized using a Tecnai G2 Spirit transmission electron microscope (FEI) at 80kv at the University of Toledo Electron Microscopy Facility.

### Multiplex cytokine analyses

Lungs, livers, and spleens were harvested from mice infected with wild-type LVS, Δ*fmvA*, *or* Δ*fmvB* on days 2 and 5 post-infection as described above. Lungs, livers, and spleens also were harvested from age-matched, negative control mice that were not infected. Cytokine concentrations from tissue homogenates were determined using the Bio-Plex Pro Mouse Cytokine 23-plex Assay (Bio-Rad), which included the following cytokines: Eotaxin, G-CSF, GM-CSF, IFN-γ, IL-1α, IL-1β, IL-2, IL-3, IL-4, IL-5, IL-6, IL-9, IL-10, IL-12 (p40), IL-12 (p70), IL-13, IL-17A, KC, MCP-1 (MCAF), MIP-1α, MIP-1β, RANTES, and TNF-α. Cytokine assays were analyzed using the with Bio-Plex 200 (Bio-Rad) system following the manufacturer’s instructions. Differences in cytokine expression were calculated by comparing averages between groups of mice infected with wild-type LVS and Δ*fmvA or* Δ*fmvB*, after having subtracted average cytokine expression from non-infected mice. Cytokines were quantitated from two independent experiments of similar design.

### *In vitro* infections of mouse bone marrow derived macrophages and HepG2 cells

Bone marrow derived macrophages were isolated from female C3H/HeN mice by first euthanizing the mice by CO_2_ asphyxiation. Femurs and tibias were aseptically harvested from both back legs and bone marrow was flushed from each bone with RPMI-1640 (Hyclone) containing 10% heat inactivated fetal bovine serum (HI-FBS, Gibco) using a 10 ml syringe with 23 gauge needle. Isolated bone marrow was gently homogenized by passing the solution through the same syringe and needle and cells were cultured in RPMI-1640 containing 10% HI-FBS and 30% day 7 L929 supernatants (mouse adipose cell line, ATCC) for 4 days at 37°C with 5% CO_2_. L929 supernatants were prepared by culturing L929 cells in RPMI containing 10% HI-FBS for either 7 days (day 7 L929 supernatant) or 14 days (day 14 L929 supernatant) at 37°C with 5% CO_2_. The day 7 and day 14 L929 supernatants were passed through a 0.22 μM filter to remove residual cells, aliquoted, and stored at -80°C until use. After isolated bone marrow cells had been incubated for 4 days, the culture medium was removed and replaced with RPMI containing 10% HI-FBS and 30% day 14 L929 supernatant. Following 2 additional days of incubation, culture medium was removed, cells were harvested by gentle scraping, cells were enumerated using a hemocytometer, 1 X 10^5^ cells in RPMI containing 10% HI-FBS were seeded into individual wells of 24-well plates, and plates were incubated overnight at 37°C with 5% CO_2_. HepG2 cells (human hepatocellular carcinoma cell line, ATCC) were cultured overnight at 37°C with 5% CO_2_ in DMEM (Hyclone) containing 10% HI-FBS and 1 X 10^5^ cells were seeded into individual wells of 24-well plates the day before bacterial infections. Bone marrow derived macrophages were infected with a multiplicity of infection (MOI) of 10 bacteria (wild-type or mutant *F*. *tularensis*) per cell (10:1). HepG2 cells were infected with an MOI of 50:1. Bone marrow derived macrophage infections were performed using RPMI-1640 containing 10% HI-FBS and HepG2 infections were performed using DMEM containing 10% HI-FBS. Following addition of *F*. *tularensis*, plates were centrifuged at 3,500 X *g* for 10 min at 10°C to initiate bacterial contact with the cells. The infected cells were incubated at 37°C with 5% CO_2_ for 2 h, washed three times with RPMI-1640 or DMEM, incubated in medium containing 100 mg/L gentamicin for 1 h to eliminate extracellular bacteria, washed three times with medium, and cells were incubated for either 2 h or 16 h in fresh medium containing 10% HI-FBS at 37°C with 5% CO_2_. Cells were lysed at 2 h and 16 h by addition of 100 μl/well of 1% saponin, cells were incubated for 20 seconds with saponin, 100 μl/well of PBS was added to each well to aid in bacterial recovery, cell lysates were serially-diluted in PBS, plated onto sMHA plates, and plates were incubated at 37°C with 5% CO_2_ for 72 h before bacterial colonies were enumerated.

### RNA sequencing sample preparation

Wild-type LVS and Δ*fmvB* were cultured in triplicate in magnesium-limiting medium as described above. Following 24 h of growth, bacteria were harvested by centrifugation, RNA was extracted and purified as described above (see RNA purification and quantitative RT-PCR), and RNA sequencing was performed by the Biomedical Genomics Core (BGC) of the Research Institute at Nationwide Children's Hospital, Columbus, Ohio. RNA sequencing data analysis was performed at the BGC with changes in Δ*fmvB* gene expression calculated based on LVS gene expression. The complete dataset from RNA sequencing is available in Table D in S2 File.

### Statistics

Differences in qRT-PCR gene expression (from infected mice and metal-limiting media) were calculated by one-way ANOVA using GraphPad Prism6 software. Differences in LPS expression (densitometry from immunoblots and flow cytometry analysis) were calculated by ImageJ (imagej.nih.gov) and FlowJo, respectively. Differences in bacterial morphology from electron microscopy images were calculated by Poisson regression analysis using R software (www.r-project.org). Differences in median time-to-death and percent survival following *F*. *tularensis* infection of mice were calculated using the log-rank Mantel-Cox test in GraphPad Prism6. Differences in lung, liver, and spleen bacterial burdens from infected mice were log10 transformed to justify the assumption of normality, then calculated by one-way ANOVA using GraphPad Prism6. Differences in cytokine expression between infection groups were calculated by two-way ANOVA using R software. All data from qRT-PCR, LPS expression, bacterial morphology, bacterial tissue burdens, and cytokine quantitation experiments are presented in bar graphs with means and standard error of the mean. *P* values of < 0.05 were considered statistically significant. As noted above, all RNA sequencing data analysis was performed by the BGC and adjusted p-values (padj) were calculated for all transcripts using DESeq2 (http://www.bioconductor.org/packages/release/bioc/html/DESeq2.html).

## Results

### FmvA and FmvB are paralogs of known *F*. *tularensis* iron acquisition genes

**Fig 1:**
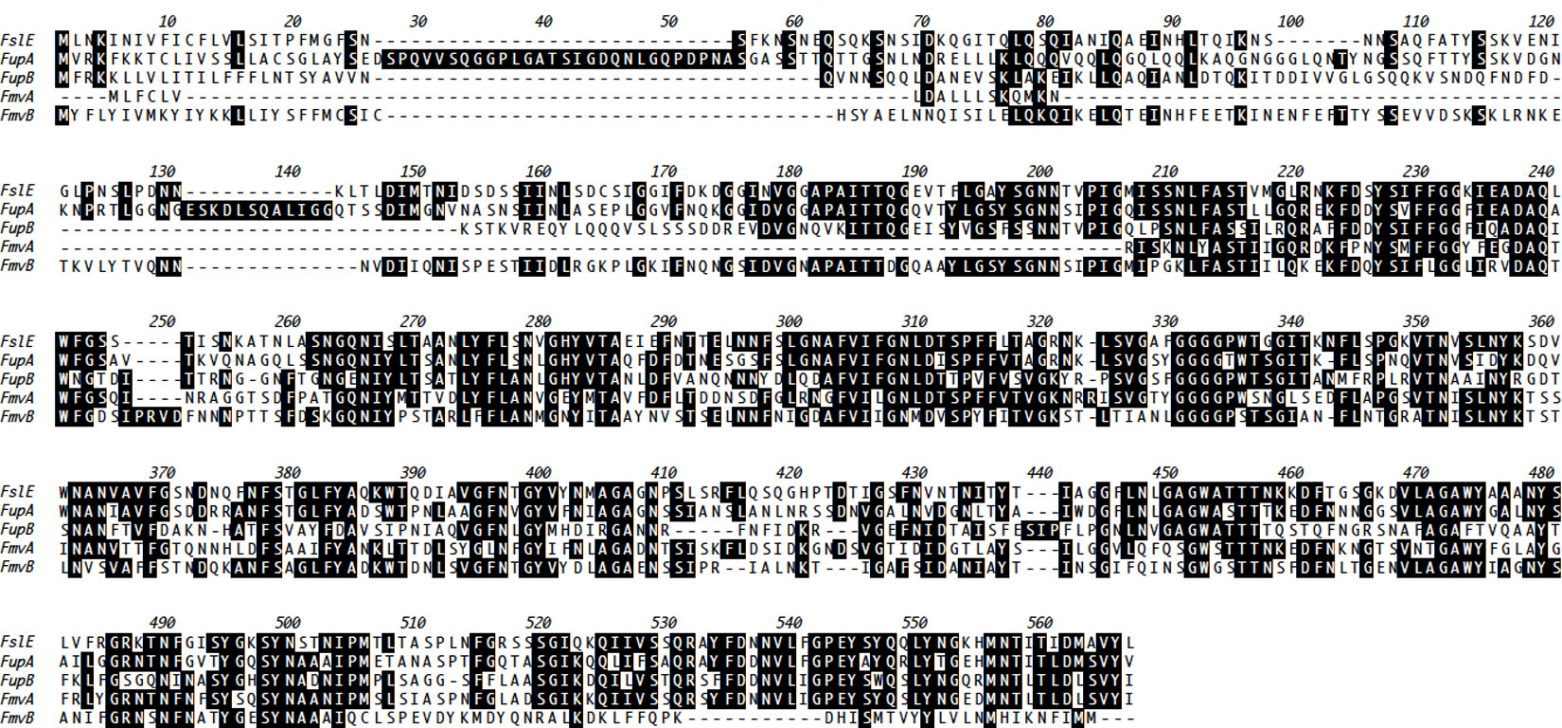
Amino acid alignment of FslE, FupA, FupB, FmvA, and FmvB. ClustalW amino acid sequence alignment for FslE (FTT0025c), FupA (FTT0918), FupB (FTT0919), FmvA (FTT0267), and FmvB (FTT0602c). Black boxes with white amino acid letters indicate conserved residues.

As with many other pathogenic bacteria [40], iron has been found to be extremely important for *F*. *tularensis* survival inside host cells [23, 24, 26, 41]. Indeed, *F*. *tularensis* has been reported to possess at least 3 iron acquisition proteins, including FslE (FTT0025c), FupA (FTT0918), and FupB (FTT0919) [23, 25, 26, 28]. In addition to iron acquisition, FupA has been shown to be required for *F*. *tularensis* virulence in macrophages and mice [22, 23]. During genomic sequencing of the highly-virulent SchuS4 strain, the above noted iron acquisition genes and two related genes, FTT0267 (designated herein as Francisella metal and virulence protein A; FmvA) and FTT0602c (designated herein as FmvB), were believed to represent a new protein family, but the true function of FmvA and FmvB has not been determined [26]. Here, the coding sequences for each of the 5 *F*. *tularensis* paralogs (FslE, FupA, FupB, FmvA, and FmvB) were compared, demonstrating between 37% and 54% amino acid identity among each paralog (Fig 1 and Table B in S1 File). Based on the amino acid identities among the paralogs and the previously published roles of FslE, FupA, and FupB in iron acquisition, we reasoned that FmvA and FmvB also may be involved iron acquisition.

Given that other Gram-negative bacterial iron acquisition systems typically are located in the outer membrane [42, 43] and our previous studies demonstrating outer membrane localization of FslE, FupA, and FupB in *F*. *tularensis* [36], we used six bioinformatics programs to predict the location and structure of FmvA and FmvB (Table 1). First, PSORTb, the bacterial protein subcellular prediction program, was used to predict the subcellular localization of FmvA and FmvB, with scores > 7.5 considered significant [44]. Whereas the localization of FmvA could not be determined by PSORTb, FmvB was predicted to be an outer membrane protein by PSORTb with a score of 9.49 (Table 1). Considering the fact that beta barrel proteins are exclusively localized to the outer membrane of Gram-negative bacteria and many metal transporters contain beta barrel structures [43, 45], we next used a series of bioinformatics programs to predict beta barrel structures in FmvA and FmvB. Second, we used PROFtmb to predict beta barrel structures in FmvA and FmvB, with *z*-values > 6 considered significant (> 40% accuracy) [46]. Using PROFtmb, FmvA was predicted to contain 14 beta strands with a *z*-value of 8.5 (71% accuracy; Table 1) and FmvB was predicted to contain 18 beta strands with a *z*-value of 6.4 (46.4% accuracy; Table 1). Third, BOMP, the beta-barrel transmembrane beta-barrel prediction program, was used to predict beta barrel structures in FmvA and FmvB, with BOMP scores > 2 (20 − 40% accurate) considered significant [47]. BOMP analysis indicated that only FmvA was a likely beta barrel protein, with a score of 3 (40 − 60% accuracy; Table 1), whereas FmvB was not predicted to contain beta barrel strands (score of 1; 0 − 20% accuracy; Table 1). Fourth, TMBB-DB was used to predict beta barrel structures in FmvA and FmvB, with scores > 0.28 considered significant [48]. Using TMBB-DB, both FmvA and FmvB were predicted to be beta barrel proteins, with a score of 1 for FmvA and a score of 0.994 for FmvB (Table 1). Fifth, TMBB-Hunt was used to predict beta barrel structures in FmvA and FmvB, with scores > 2 considered significant [49]. Using TMBB-Hunt, FmvA had a beta barrel score of 10.63 and FmvB had a beta barrel score of 7.45, indicating that both proteins are likely beta barrels (Table 1). Finally, in conjunction with the above noted beta barrel predictions, FmvA and FmvB beta barrel topologies were modeled using the PRED-TMBB program and visualized by TMRPres2D (Fig A in S1 File). PRED-TMBB modeling indicated that FmvA contains 16 beta strands and 8 extracellular loops, including 3 loops which are > 20 amino acids in length. In contrast, PRED-TMBB modeling indicated that FmvB contains 21 beta strands and 10 extracellular loops, including 6 loops which are > 20 amino acids in length. The large extracellular loops in both FmvA and FmvB (Fig A in S1 File) indicate that these proteins may play a role in transport processes, as outer membrane transport proteins in other bacteria are known to contain large extracellular loops [50]. Three-dimensional structural predictions of FmvA and FmvB were generated using TMBpro (Fig A in S1 File). For FmvA, 8 beta strands were predicted and for FmvB 9 beta strands were predicted. However, for both proteins, TMBpro predicted long N-terminal unstructured regions (Fig A in S1 File) that likely contributed to fewer predicted beta strands than were predicted by either PROFtmb or PRED-TMBB. Taken together, 6 out of 7 bioinformatics programs indicated that FmvA is a beta barrel protein and 6 out of 7 bioinformatics programs indicated that FmvB is an outer membrane-localized beta barrel protein.

**Table 1:** FmvA and FmvB are predicted outer membrane beta barrel proteins.

Protein	PSORTb localization and score^a^	PROFtmb score (β strands)^b^	BOMP score^c^	TMBB-DB score^d^	TMB-Hunt score^e^
FmvA	Un 0.11	8.5 (14 β strands)	3	1.0	10.63
FmvB	OM 9.49	6.4 (18 β strands)	1	0.994	7.45

^a^ PSORTb version 3.0.2 bacterial protein subcellular localization prediction program (http://www.psort.org/psortb/). Outer membrane (OM) or unknown (Un) localization. Scores > 7.5 considered significant.

^b^ PROFtmb bacterial transmembrane beta-barrel prediction program (http://cubic.bioc.columbia.edu/services/proftmb/). *z-*value scores > 4 considered significant. Number of β strands predicted by PROFtmb indicated in parentheses.

^c^ BOMP beta-barrel integral outer membrane protein prediction program (http://www.bioinfo.no/tools/bomp). Scores > 2 considered significant.

^d^ TMBB-DB transmembrane beta-barrel prediction program (http://beta-barrel.tulane.edu/). Scores > 0.28 considered significant.

^e^ TMB-Hunt transmembrane beta-barrel prediction program (http://bmbpcu36.leeds.ac.uk/~andy/betaBarrel/AACompPred/aaTMB_Hunt.cgi). Scores > 2 were significant.

### *fmvB* is up-regulated during mammalian infection

**Fig 2:**
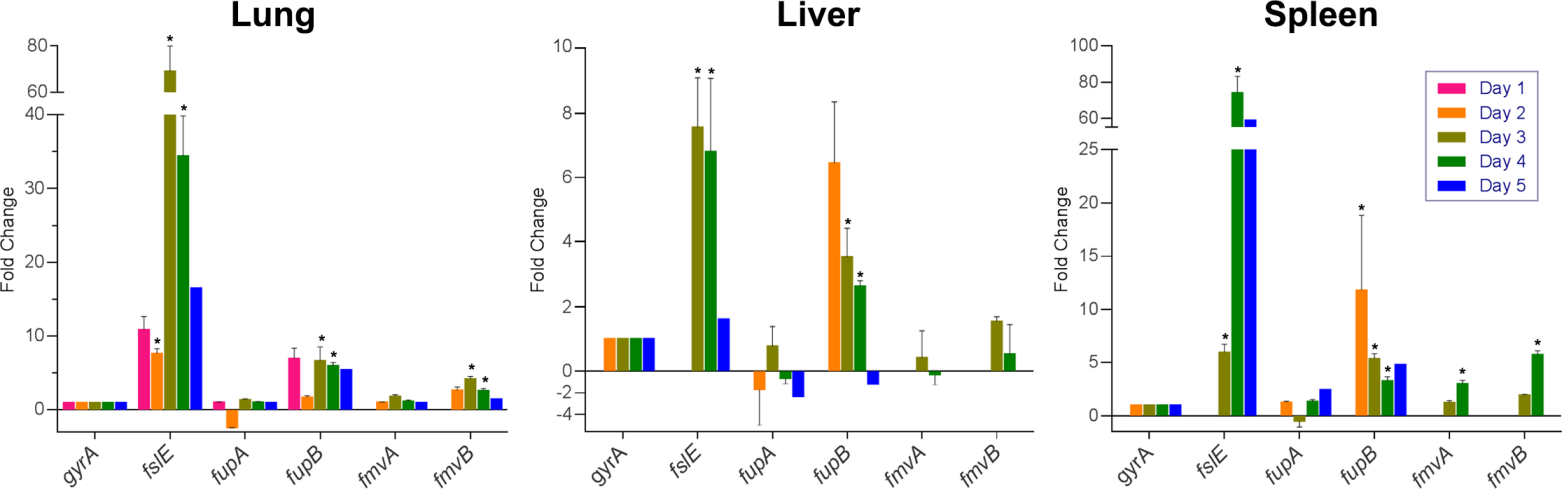
Gene expression analysis from *F*. *tularensis* SchuS4-infected mice. Groups of 4 female C3H/HeN mice were intranasally-infected with 30 CFU of *F*. *tularensis* strain SchuS4. Lungs, livers, and spleens were harvested from days 1 through 5 post-infection. qRT-PCR was performed to quantitate the relative gene expression of *fslE*, *fupA*, *fupB*, *fmvA*, and *fmvB* during *in vivo* infection compared to SchuS4 grown in laboratory medium. Data were normalized to ‘housekeeping’ gene DNA gyrase α subunit (*gyrA*; FTT1575c). Significant differences are indicated by * (*P* < 0.05).

Studies to identify bacterial virulence factors typically have focused on common disease-causing molecules/pathways, including toxins, adhesins, proteases, antibiotic resistance proteins, and multi-component systems for secretion and motility [51–53]. However, given that *F*. *tularensis* lacks many of these common virulence factors, there is a clear need to identify *F*. *tularensis* virulence factors by other means. Indeed, it is now well-appreciated that transcriptional analyses of bacteria during *in vivo* infections can lead to the discovery of new or non-conventional virulence factors [54–56]. The premise for such *in vivo* transcriptional analyses is that, compared with bacterial transcripts from laboratory-grown bacteria, bacterial genes up-regulated during mammalian infections likely are virulence factors. Given that FslE, FupA, and FupB previously were shown to play roles in *F*. *tularensis* iron acquisition [23, 25, 26, 28], FupA was shown to be required for *F*. *tularensis* virulence [22, 23], and FslE, FupA, FupB, FmvA, and FmvB share between 37% and 59% amino acid identity (Fig 1 and Table B in S1 File), we assessed gene expression changes for all five paralogs (*fslE*, *fupA*, *fupB*, *fmvA*, and *fmvB*) from *F*. *tularensis-*infected mouse tissues to correlate FmvA or FmvB with iron acquisition and/or virulence. Mice were intranasally infected with 30 CFU of *F*. *tularensis* SchuS4, with lungs, livers, and spleens harvested once per day for five days following infection, and quantitative real-time PCR (qRT-PCR) analysis was performed to examine gene expression changes for *fslE*, *fupA*, *fupB*, *fmvA*, and *fmvB* during mouse infection, compared with SchuS4 from laboratory growth medium. Despite the previously-reported role of FslE in *F*. *tularensis* iron acquisition and virulence, *in vivo fslE* gene expression had not been previously examined. Of the five paralogs examined, *fslE* was the most upregulated paralog in all tissues examined, including 7.6- to 69.2-fold upregulation in the lung, 1.6- to 7.5-fold upregulation in the liver, and 6.0- to 74.2-fold upregulation in the spleen, depending on the day post-infection (Fig 2). These results are interesting because, although previous *in vitro* studies noted that *fslE* was upregulated approximately 2-fold in iron-limiting conditions [24] and played an important role in SchuS4 iron acquisition [23, 25], the dynamic *in vivo* upregulation of *fslE* demonstrates that iron uptake is critical for *F*. *tularensis* survival and replication inside the host and suggests that *fslE* may respond to factors other than iron in the complex mammalian environment. By comparison, *fupA* expression was relatively unchanged (-2.4- to 2.4-fold change) in lungs, livers, and spleens of infected mice at all time points examined (Fig 2), indicating that *fupA* may be constitutively expressed in SchuS4. These *in vivo* results confirm previous *in vitro* analysis noting no change in *fupA* expression in iron limitation [24]. Given that FupA has been reported to be important for SchuS4 iron acquisition [23], it is entirely possible that *fupA* is expressed at constant levels to ensure consistent intracellular iron levels for various bacterial processes, similar to constitutive iron transporters reported for other pathogenic bacteria [57]. Next, *fupB* was found to be moderately upregulated (1.2- to 11.8-fold) in lungs, livers, and spleens of infected mice from days 3 through 5 post-infection (Fig 2). These results were surprising, given that FupB is not involved in SchuS4 virulence [22] and is not required for iron acquisition in SchuS4 [23]. Expression of *fmvA* only was found to be significantly upregulated (3.0-fold) in infected spleens on day 4 post-infection, despite a trend towards upregulation in lungs and spleen on day 3 post-infection. By comparison, *fmvB* was found to be significantly upregulated in infected lungs on days 3 and 4 post-infection (4.2-fold and 2.6-fold, respectively) and in infected spleens (5.8-fold) on day 4 post-infection (Fig 2). Based on homology of FmvA and FmvB to FslE, FupA, and FupB, and the previously-reported role of FslE and FupA in virulence and iron acquisition, these *in vivo* expression data indicated that FmvA and FmvB also may play roles in *F*. *tularensis* virulence and/or iron uptake.

For safety considerations, we decided to perform all subsequent experiments using *F*. *tularensis* Type B strain LVS (can be safely manipulated at BSL2). Although the genomes of SchuS4 and LVS share greater than 98% sequence identity, the two *F*. *tularensis* strains differ in their ability to cause human disease. As such, we examined gene expression levels of *fslE*, *fupAB*, *fmvA*, and *fmvB* from mice infected with LVS to more directly compare our gene expression data from SchuS4 (Fig 2) with subsequent studies in LVS (below). Mice were intranasally infected with 6 ✕ 10^4^ CFU of wild-type LVS and lungs, livers, and spleens were harvested on day 2 and day 5 post-infection. qRT-PCR analysis was performed to examine gene expression changes for *fslE*, *fupAB*, *fmvA*, and *fmvB* during mouse infection, compared with LVS from laboratory growth medium (Fig B in S1 File). Although LVS *fslE* expression was not significantly altered on day 2 post-infection, LVS *fslE* was upregulated in all three tissues on day 5 post-infection (30-fold upregulated in lungs, 5-fold upregulated in livers, and 11-fold upregulated in spleens; Fig B in S1 File), consistent with findings in SchuS4-infected mice (Fig 2). Expression of LVS *fupAB*, the result of a gene fusion event between *fupA* and *fupB*, was not significantly changed in lungs, livers, or spleens of infected mice (Fig B in S1 File), which was consistent with our data for SchuS4 *fupA* expression in infected mice (Fig 2). LVS *fmvA* was upregulated approximately 3-fold in the lungs, livers, and spleens of infected mice on day 5 post-infection (Fig B in S1 File), which generally followed the same trends observed in SchuS4-infected mice (Fig 2). Finally, LVS *fmvB* was found to be 3.4-fold upregulated in the liver on day 5 post-infection (Fig B in S1 File), whereas SchuS4 *fmvB* was upregulated in lungs and spleens (Fig 2). Taken together, these data suggest that the SchuS4 and LVS gene paralogs respond similarly to environmental cues.

### *fmvB* is upregulated in Mg limitation

**Fig 3:**
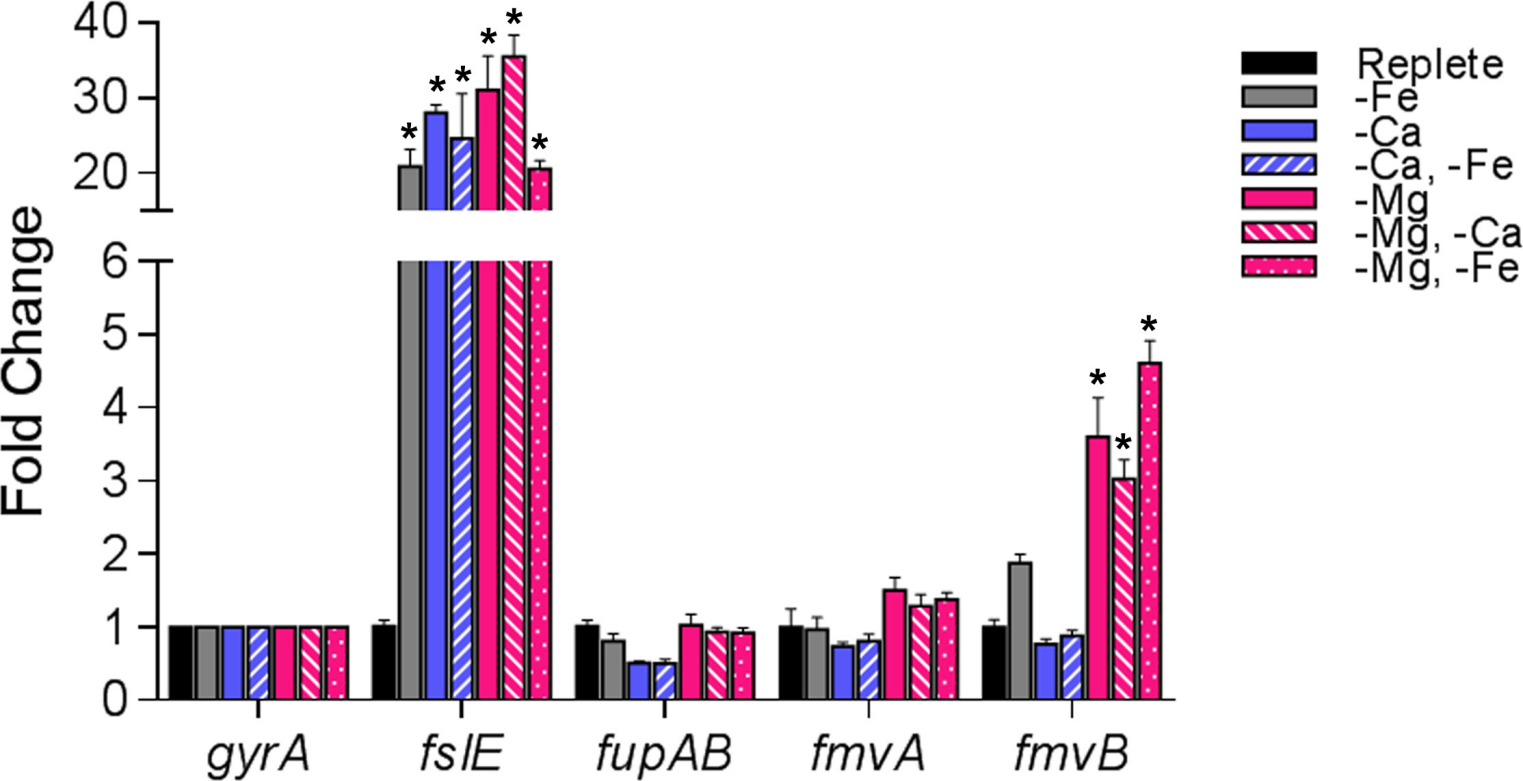
Transcriptional changes of *F*. *tularensis fmvA and fmvB* paralogs in metal-depleted media. *F*. *tularensis* LVS was grown in either CDM supplemented with iron, calcium, and magnesium (replete) or metal-depleted CDM containing magnesium and calcium (low iron; -Fe), magnesium and iron (no calcium; -Ca), iron and calcium (no magnesium; -Mg), magnesium only (no calcium and low iron; -Ca, -Fe), iron only (no magnesium or calcium; -Mg, -Ca), or calcium only (no magnesium and low iron; -Mg, -Fe). qRT-PCR was performed to quantitate the relative gene expression of *fslE*, *fupAB*, *fmvA*, and *fmvB* in each metal-depleted medium. Data were normalized to ‘housekeeping’ gene DNA gyrase α subunit (*gyrA*; FTL0533). Significant differences are indicated by * (*P* < 0.05).

Whereas *fslE*, *fupA*, *and fupB* previously have been shown to play roles in *F*. *tularensis* iron uptake, the functions of *fmvA* and *fmvB* are unknown. Based on homology to *fslE*, *fupA*, *and fupB*, it generally had been assumed that *fmvA* and *fmvB* also function in iron acquisition [21, 26]. However, studies in other bacteria have demonstrated that metal uptake systems, including those for copper, magnesium, and zinc, often share sequence homology with iron acquisition systems [57–59]. Thus, we took an unbiased approach to determine if *fmvA* and *fmvB* are responsive to various metal-limiting conditions by measuring *F*. *tularensis* LVS gene expression changes for *fmvA*, *fmvB*, *fslE*, and *fupAB* by qRT-PCR. Given that chemically-defined medium (CDM; [25, 26, 35]) and Mueller Hinton (MH; [23, 29, 30]) medium, routinely used for *F*. *tularensis* growth, both require magnesium, calcium, and iron supplementation, we focused our efforts on gene expression changes in magnesium, calcium, and iron limitation. LVS was grown in either chemically-defined medium (CDM) with supplemented iron, calcium, and magnesium (CDM replete) or metal-depleted CDM containing supplemented magnesium and calcium (low iron; -Fe), magnesium and iron (no calcium; -Ca), magnesium only (no calcium and low iron; -Ca, -Fe), iron and calcium (no magnesium; -Mg), iron only (no magnesium or calcium; -Mg, -Ca), or calcium only (no magnesium and low iron; -Mg, -Fe). *fslE*, previously reported to be an outer membrane protein that plays a secondary role in LVS siderophore-dependent iron acquisition [26, 28, 36], was dramatically upregulated (17.8- to 40.5-fold) in all metal-limiting conditions tested (Fig 3), suggesting that *fslE* may be involved in uptake of iron, calcium, and magnesium. By comparison, *fupAB*, previously reported to be an outer membrane protein that is the primary iron acquisition system for LVS [26, 28, 36], was virtually unchanged (0.5- to 1-fold) in all metal-limiting conditions tested (Fig 3). Because *fupAB* was reported to be the dominant uptake system for both siderophore-dependent ferric iron and siderophore-independent ferrous iron in LVS [28], the lack of any *fupAB* expression changes in response to various metal-limiting conditions was surprising and suggests that *fupAB* may be constitutively expressed in LVS. Similarly, *fmvA* expression was not significantly changed (0.8- to 1.5-fold) in response to various metal-limiting conditions (Fig 3). Given the lack of previous studies on *fmvA*, we do not know if *fmvA* is constitutively expressed or if *fmvA* has a function unrelated to iron, calcium, or magnesium uptake. Interestingly, *fmvB* was significantly upregulated in three different magnesium-limiting conditions: CDM supplemented with iron and calcium (no magnesium; -Mg; *fmvB* upregulated 3.5-fold), CDM supplemented with iron only (no magnesium or calcium; -Mg, -Ca; *fmvB* upregulated 3-fold), and CDM supplemented with calcium only (no magnesium or iron; -Mg, -Fe; *fmvB* upregulated 4.6-fold; Fig 3). However, *fmvB* expression was unchanged in the other metal-limiting conditions tested (*e*.*g*. no iron; no calcium, etc.; Fig 3). Taken together, despite homology of the previously unstudied *F*. *tularensis fmvA* and *fmvB* genes to the iron acquisition genes *fslE* and *fupAB*, *fmvA* and *fmvB* do not appear to be regulated by iron. Instead, these data indicate that *fmvB* may play a role in magnesium acquisition.

### Δ*fmvA* and Δ*fmvB* mutants are not defective for growth in iron-limiting conditions

Although *fupAB* has been reported to be the dominant uptake system for both siderophore-dependent ferric iron and siderophore-independent ferrous iron in LVS [28], our transcriptional analyses found that *fupAB* expression was virtually unchanged in iron-limiting conditions (Fig 3). These results suggest that gene expression changes are not necessarily correlated with roles in iron uptake and, thus, we could not exclude the possibility that FmvA and FmvB may play roles in iron uptake based solely on qRT-PCR gene expression data. To more directly examine the role of FmvA and FmvB in *F*. *tularensis* iron uptake, we generated Δ*fmvA* and Δ*fmvB* mutants in LVS and examined their growth in iron-replete and iron-limiting conditions (Fig C in S1 File). Similar numbers of wild-type LVS, Δ*fmvA*, Δ*fmvB*, or Δ*fupAB* (control; previous studies demonstrated that Δ*fupAB* was defective for growth in iron-limiting conditions; [26]) bacteria were inoculated into either CDM replete liquid medium or metal-depleted CDM liquid medium supplemented with magnesium and calcium (low iron; -Fe) and bacterial growth was measured over the course of 50 h. In CDM replete liquid medium, wild-type LVS and the three mutants grew similarly over the course of 50 h (Fig C in S1 File), demonstrating that there was no inherent growth defect in any of the mutants. By comparison, in CDM liquid medium containing low iron (-Fe), only the Δ*fupAB* mutant was defective for growth (Fig C in S1 File). To confirm that these results were not liquid medium-dependent, we also grew wild-type LVS and the three mutants on CDM replete agar or CDM agar containing low iron (-Fe; Fig C in S1 File). Similar to results in liquid media, only the Δ*fupAB* mutant was defective for growth on iron-limiting agar (Fig C in S1 File).

Although the above results suggested that FmvA and FmvB may not be involved in LVS iron uptake, previous studies indicated that there may be compensation or cooperation among the five paralogs, which could mask the function of individual paralogs. More specifically, Sen *et al*. found that when LVS *ΔfslE* was grown in iron-limiting medium, no growth defect was observed. Conversely, when LVS *ΔfupAB* was grown in iron-limiting medium, a significant growth defect was observed. Interestingly, a *ΔfslE*/*ΔfupAB* double mutant exhibited a much more severe grown defect in iron-limiting medium than the *ΔfupAB* mutant alone [26]. Those results indicated that *fupAB* is the major iron acquisition system for LVS, that *fslE* and *fupAB* work cooperatively to import iron, and that *fupAB* is able to compensate for the loss of *fslE*. Here, to test if *fupAB* works cooperatively with either *fmvA* or *fmvB* to import iron, we generated *ΔfmvA/ΔfupAB* and *ΔfmvB/ΔfupAB* double mutants and examined the growth of the double mutants in iron-replete and iron-limiting conditions (Fig D in S1 File). In CDM replete liquid medium, wild-type LVS, Δ*fupAB*, Δ*fmvA/*Δ*fupAB*, and Δ*fmvB/*Δ*fupAB* grew similarly over the course of 52 h (Fig D in S1 File), demonstrating that there was no inherent growth defect in any of the mutants. In CDM liquid medium containing low iron (-Fe), wild-type LVS grew similarly as in CDM replete medium (Fig D in S1 File). However, in CDM liquid medium containing low iron, Δ*fupAB*, Δ*fmvA/*Δ*fupAB*, and Δ*fmvB/*Δ*fupAB* were equally deficient for growth over the course of 52 h, demonstrating that there was no additional growth defect by deleting *fmvA* or *fmvB* in a Δ*fupAB* mutant (Fig D in S1 File). To confirm that these results were not liquid medium-dependent, we also grew wild-type LVS, Δ*fupAB*, Δ*fmvA/*Δ*fupAB*, and Δ*fmvB/*Δ*fupAB* on CDM replete agar or CDM agar containing low iron (-Fe; Fig D in S1 File.). Similar to results in liquid media, Δ*fupAB*, Δ*fmvA/*Δ*fupAB*, and Δ*fmvB/*Δ*fupAB* were equally deficient for growth on CDM agar containing low iron, demonstrating that there was no additional growth defect by deleting *fmvA* or *fmvB* in a Δ*fupAB* mutant (Fig D in S1 File). Taken together, given that neither *fmvA* nor *fmvB* were upregulated in iron-limiting conditions (Fig 3), neither Δ*fmvA* nor Δ*fmvB* exhibited any growth defects in iron-limiting medium (Fig C in S1 File), and that double gene deletions of Δ*fmvA/*Δ*fupAB* and Δ*fmvB/*Δ*fupAB* did not result in additional growth defects in iron-limiting medium, as compared to Δ*fupAB* (Fig D in S1 File), it appears unlikely that FmvA and FmvB are involved in iron uptake.

### Δ*fmvA* and Δ*fmvB* mutants are not defective for growth in magnesium-limiting conditions

Given that *fmvB* gene expression was upregulated in magnesium limitation (Fig 3), we hypothesized that Δ*fmvB* would exhibit growth defects when grown in magnesium-limiting medium. To test this hypothesis and to gather additional information about the function of FmvA, we examined the growth of Δ*fmvA* and Δ*fmvB* in magnesium-replete and magnesium-limiting conditions (Fig E in S1 File). In agreement with our previous growth data from CDM replete liquid medium (Fig C in S1 File.), wild-type LVS, Δ*fmvA*, *and* Δ*fmvB* grew similarly over the course of 50 h (Fig E in S1 File). Contrary to our hypothesis, wild-type LVS, Δ*fmvA*, and Δ*fmvB* grew similarly over the course of 50 h in magnesium-limiting CDM (-Mg) (Fig E in S1 File). Next, because both *fslE* and *fmvB* were upregulated in magnesium-limiting conditions (Fig 3) and to account for any potential compensation between *fslE* and *fmvB* to import magnesium, we tested the ability of wild-type LVS, Δ*fmvB*, Δ*fslE*, *and* Δ*fmvB/*Δ*fslE* to grow in magnesium-replete and magnesium-limiting conditions (Fig F in S1 File). However, wild-type LVS, Δ*fmvB*, Δ*fslE*, and Δ*fmvB/*Δ*fslE* grew similarly over the course of 50 h in both magnesium-replete and magnesium-limiting conditions (Fig F in S1 File). Finally, to account for any potential compensation between *fupAB* and *fmvB* to import magnesium, we generated a Δ*fmvB/*Δ*fupAB* double mutant and examined the growth wild-type LVS, Δ*fmvB*, Δ*fupAB*, and Δ*fmvB/*Δ*fupAB* in magnesium-replete and magnesium-limiting conditions (Fig F in S1 File). However, all of the mutants grew similarly to wild-type over the course of 50 h in both magnesium-replete and magnesium-limiting conditions (Fig F in S1 File). Taken together, despite the fact that *fmvB* was significantly upregulated in magnesium limitation (Fig 3), *fmvB* does not appear to be required for *F*. *tularensis* LVS growth in magnesium limitation.

### FmvB is an outer membrane protein

**Fig 4:**
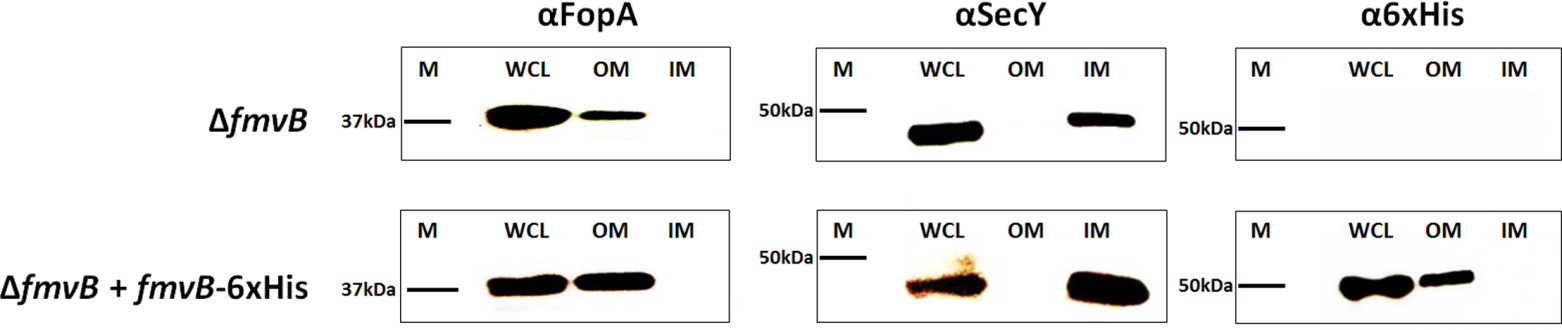
FmvB is localized to the *F*. *tularensis* outer membrane. Spheroplasting, osmotic lysis, and sucrose density gradient centrifugation were performed to separate inner membranes and outer membranes from *F*. *tularensis* Δ*fmvB* and Δ*fmvB* complemented with a C-terminal histidine-tagged FmvB *(*Δ*fmvB* + *fmvB-*6xHis). Equal amounts of whole-cell lysates (WCL), outer membrane fractions (OM), and inner membrane fractions (IM) from Δ*fmvB* and Δ*fmvB* + FmvB-6xHis were separated by SDS-PAGE, transferred to nitrocellulose, and immunoblotting was performed using antisera specific for the OM control protein FopA (αFopA), IM control protein SecY (αSecY), or the histidine tag (α6x-His). m, prestained molecular mass standards with sizes (in kDa) noted on the left side of each immunoblot.

As noted above, the majority of bioinformatics programs used here predicted that FmvB was an outer membrane (OM) protein (Table 1; Fig A in S1 File). To confirm that FmvB was OM-localized in LVS, FmvB with a C-terminal 6x histidine fusion tag was expressed in Δ*fmvB (*Δ*fmvB* + *fmvB*-6xHis) and bacteria were subjected to spheroplasting, osmotic lysis, and sucrose density gradient centrifugation as previously described [36, 60]. Equal amounts of representative sucrose gradient fractions were analyzed by immunoblotting to localize FopA (OM control; [36]), SecY (inner membrane control; [36]) and FmvB. As expected, in *ΔfmvB* FopA was localized to the OM, SecY was localized to the inner membrane (IM), and FmvB was not detected (Fig 4). In the *ΔfmvB* + *fmvB*-6xHis complemented strain, FopA was localized to the OM, SecY was localized to the IM, and FmvB was localized to the OM (Fig 4). These results confirm the bioinformatic predictions (Table 1) that *F*. *tularensis* FmvB is OM-localized.

### Δ*fmvB* does not have altered membrane integrity

Although we were unable to demonstrate that FmvB is required for the uptake of either iron or magnesium (Figs C-F in S1 File), the upregulation of *fmvB* in magnesium-limiting media (Fig 3) suggests that FmvB still may be involved in some unknown mechanism of *F*. *tularensis* magnesium sensing, magnesium regulation, or that FmvB has a function that is important during magnesium limitation. Given the well-known role of magnesium to stabilize the OM [16, 61] and our data demonstrating that FmvB was OM-localized (Fig 4), we tested whether *ΔfmvB* bacteria had altered membrane integrity by growing Δ*fmvB* in the presence of different antibiotics, detergents, dyes (Table C in S1 File), and antimicrobial peptides (Fig G in S1 File). Compared with wild-type LVS, *ΔfmvB* was not significantly more sensitive to the antibiotics gentamicin, ciprofloxacin, or tetracycline (Table C in S1 File). In addition, *ΔfmvB* was not significantly more sensitive to detergents such as SDS, Triton X-100, or CTAB than wild-type LVS (Table C in S1 File). Finally, *ΔfmvB* was not significantly more sensitive to ethidium bromide than wild-type LVS (Table C in S1 File). We also compared wild-type LVS and *ΔfmvB* sensitivity to the antimicrobial peptides LL-37, hBD-3, HNP-2, and polymyxin B, however *ΔfmvB* was no more susceptible to these four antimicrobial peptides than wild-type LVS (Fig G in S1 File). These membrane integrity studies demonstrated that Δ*fmvB* did not have increased sensitivity to any of the membrane perturbing and antimicrobial agents tested, suggesting that FmvB is not required for *F*. *tularensis* membrane integrity.

### Δ*fmvB* expresses more LPS in magnesium-limiting medium

**Fig 5:**
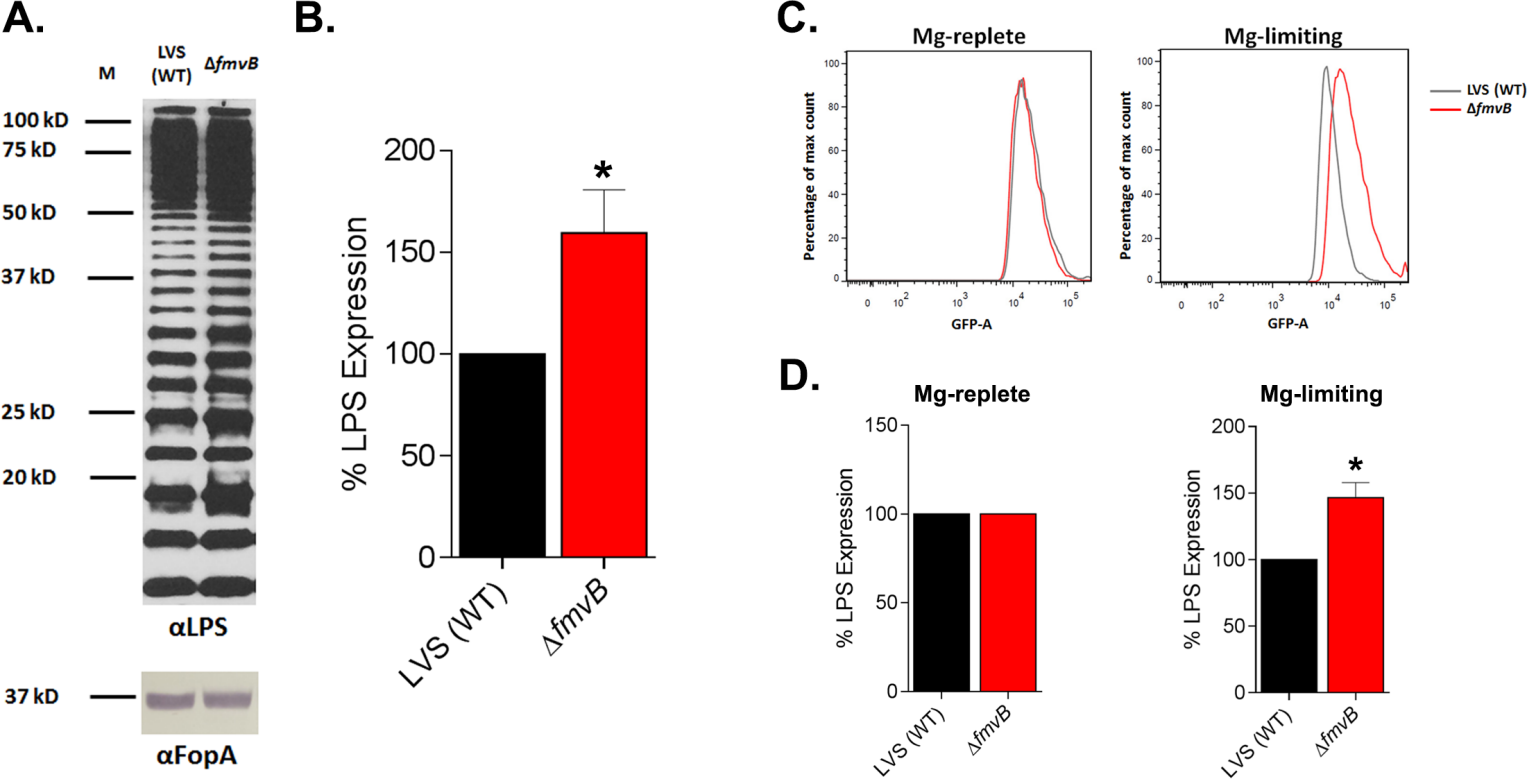
Δ*fmvB* expresses more LPS than wild-type LVS. (A) Wild-type LVS and Δ*fmvB* were cultured in magnesium-limiting medium, whole cell lysates were separated by SDS-PAGE, transferred to nitrocellulose, and immunoblotting was performed using antisera specific for either *F*. *tularensis* LPS (αLPS) or FopA (loading control; αFopA). m, molecular mass standards with sizes (in kDa) noted on the left side of each immunoblot. (B) Percent LPS expression in wild-type LVS and Δ*fmvB* was calculated, based on densitometry analysis of immunoblots. LVS LPS expression was arbitrarily set at 100% and densitometry was normalized to FopA loading controls. (C) Wild-type LVS and Δ*fmvB* from CDM replete or magnesium-limiting medium were surface labeled with αLPS then detected using GFP secondary antibody and flow cytometry analysis. (D) Percent LPS expression in wild-type LVS and Δ*fmvB* was calculated, based on flow cytometry analysis in CDM replete and magnesium-limiting medium. LVS LPS expression was arbitrarily set at 100%. Significant differences, compared to WT, are indicated by * (*P* < 0.05).

Continuing with our investigation of a potential link between upregulation of FmvB in magnesium limitation and FmvB OM localization, we compared LPS expression in wild-type LVS and *ΔfmvB*, given the well-known role of magnesium to neutralize LPS negative charges and the ability of many bacteria to alter their LPS in low magnesium [62]. First, we compared the LPS ‘ladder-like’ pattern of wild-type LVS and Δ*fmvB* whole cell lysates grown in magnesium-limiting medium by immunoblotting and detected no observable differences in repeating carbohydrate banding patterns between the two strains (Fig 5A). However, we did consistently observe that Δ*fmvB* expressed more total LPS than wild-type LVS, particularly in the higher molecular weight range (*e*.*g*. 50 to 100 kDa; Fig 5A). To quantitate LPS differences between Δ*fmvB* and wild-type LVS, densitometry analysis from 4 independent experiments was performed, demonstrating that Δ*fmvB* expressed approximately 50% more LPS than wild-type LVS (Fig 5B). Next, to confirm increased LPS expression in Δ*fmvB* and to determine if this increased LPS production was localized to the bacterial surface, we quantitated *F*. *tularensis* whole bacterial LPS surface expression by flow cytometry (Fig 5C and 5D). In replete liquid medium, wild-type LVS and Δ*fmvB* expressed nearly equivalent amounts of LPS on their surface (Fig 5C and 5D). In contrast, in magnesium-limiting liquid medium, Δ*fmvB* expressed 50% more LPS than wild-type LVS (Fig 5C and 5D), in agreement with our immunoblot data (Fig 5A and 5B). Taken together, two different analyses demonstrated that Δ*fmvB* expressed approximately 50% more LPS than wild-type LVS in magnesium-limiting medium and this increased LPS was located on the surface of the bacterium. Although we do not understand the exact mechanism by which FmvB controls LPS levels on the bacterial surface, our data suggest that *F*. *tularensis* requires FmvB to limit LPS surface expression during magnesium-limiting conditions.

### Δ*fmvB* has altered cell morphology in magnesium-limiting medium

**Fig 6:**
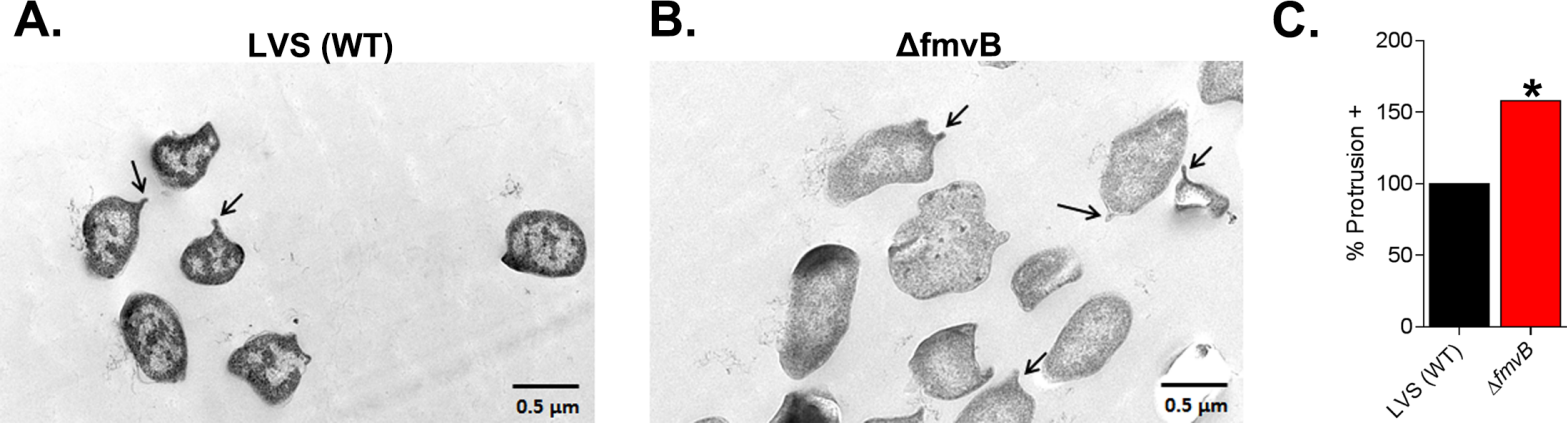
Δ*fmvB* bacteria have increased numbers of protrusions in magnesium-limiting medium. Wild-type LVS and Δ*fmvB* were cultured in magnesium-limiting medium and visualized using transmission electron microscopy. (A) Representative images of bacterial surface protrusions; (B) Approximately 200 wild-type and Δ*fmvB* bacteria were examined, protrusions were counted, and the percentages of protrusions were calculated for each bacterial strain. LVS was arbitrarily set at 100%. Significant differences, compared to WT, are indicated by * (*P* < 0.05).

Because Δ*fmvB* expresses 50% more LPS than wild-type LVS (Fig 5) and no defects were observed in Δ*fmvB* outer membrane integrity (Table C in S1 File; Fig G in S1 File), we speculated that Δ*fmvB* might be generating membrane blebs or vesicles at the bacterial surface to help offset increased LPS. Thus, we examined the morphology of wild-type LVS and Δ*fmvB* bacteria grown in magnesium-limiting medium by transmission electron microscopy (TEM). As demonstrated in representative images (Fig 6), whereas approximately 5 to 10% of wild-type LVS bacteria possessed surface protrusions (Fig 6A), increased percentages (approximately 10 to 15%) of Δ*fmvB* bacteria had surface protrusions (Fig 6B). We examined multiple fields by TEM and counted the number of protrusions for over 200 LVS or Δ*fmvB* bacteria, finding that Δ*fmvB* bacteria expressed 50% more surface protrusions than wild-type LVS (Fig 6C).

### RNAseq analysis of Δ*fmvB* in low Mg

To better understand the mechanisms by which FmvB is upregulated in magnesium limitation and to explain why Δ*fmvB* expresses 50% more LPS on its surface than wild-type LVS, we analyzed whole genome transcripts from LVS and Δ*fmvB* grown in magnesium limitation by RNA sequencing. For the purposes of this study, we focused our attention on transcriptional changes linked to the bacterial envelope, including changes in inner membrane proteins, outer membrane proteins, LPS/capsule synthesis, stress responses, and genes involved in metal uptake/sensing (Table 2). Interestingly, *kdtA* (required for core oligosaccharide synthesis;[63]) and *wbtA* (required for *F*. *tularensis* capsule and LPS production; [64]) were found to be upregulated 1.56- and 1.47-fold (Table 2), respectively, in Δ*fmvB*, possibly explaining the 50% LPS increase in Δ*fmvB* (Fig 5). However, FTL1420 and FTL1432, linked to a glycoprotein capsule-like complex loci in LVS, were found to be downregulated 1.39- to 1.36-fold in Δ*fmvB* (Table 2). As recently reviewed [65], although a majority of evidence indicates that the *F*. *tularensis* capsule is composed of O-antigen and many LPS synthesis genes are required for capsule production, there also have been reports of a glycoprotein capsule-like complex, possibly linked to FTL1420 and FTL1432. Regardless of this controversy, the roles of *kdtA* and *wbtA* in *F*. *tularensis* LPS synthesis are well-established. The upregulation of various *F*. *tularensis* genes encoding hypothetical inner membrane proteins, stress response proteins, or metal uptake/sensing proteins in Δ*fmvB* (Table 2) would be interesting to speculate on, but at this time, little is known about these proteins. Only *fslB* (FTL1883) and *fslD* (FTL1835), which are in the *fslE* operon and are predicted inner membrane proteins (data not shown), have been examined at a cursory level in a previous publication [25]. Of the various genes down-regulated in Δ*fmvB*, *lpxE* (lipid A 1’ phosphatase) is most interesting because reduced expression of *lpxE* would be predicted to result in hyper-phosphorylation of LPS in Δ*fmvB* and potentially would increase immunogenicity of Δ*fmvB*. However, *lpxE* down-regulation was marginal (1.4-fold; Table 2) and we do not know if decreased *lpxE* expression in Δ*fmvB* contributed to the observed attenuated of Δ*fmvB* in mice (see next section on mouse infections). In addition, hypothetical outer membrane lipoprotein VacJ (FTL0765) was found to be downregulated 1.46-fold in Δ*fmvB* (Table 2). Little is known about *F*. *tularensis* VacJ, although a previous study noted that two VacJ homologs, including FTL0765, exist in LVS [66]. *Shigella flexneri* VacJ is a surface exposed lipoprotein that has been shown to be required for *S*. *flexneri* to invade host cells, possibly through a membrane rupturing mechanism [67]. Interestingly, *Actinobacillus pleuropneumoniae*, a pulmonary pathogen of swine, encodes a VacJ homolog that has been shown to play a role in membrane integrity, serum resistance, and biofilm formation [68]. When tested, we did not observe any host cell invasion (described below) or membrane integrity defects for Δ*fmvB* (Fig G in S1 File and Table C in S1 File). However, future studies may be warranted to examine the role of VacJ in *F*. *tularensis* virulence. Finally, hypothetical outer membrane lipoproteins FTL1372 and FTL0765 also were found to be down-regulated 1.76- and 1.46-fold, respectively, in Δ*fmvB* (Table 2). At this time, it is premature to speculate if down-regulation of FTL1372 and FTL0765 contributed to the observed attenuation of Δ*fmvB* in mice (see next section on mouse infections), but further studies are needed to better understand these findings.

**Table 2:** RNA sequencing analysis of transcripts up- or down-regulated in Δ*fmvB*.

LVS locus	SchuS4 homolog	Classification^a^	Description	Fold-change (relative to LVS)
FTL1199	FTT1001	Metal	Fe^2+^ or Zn^2+^ regulatory protein similar to Fur	1.79
FTL0919	FTT0646c	IM	Hypothetical membrane protein	1.77
FTL1833	FTT0028c	Metal	FslB; major Facilitator Superfamily (MFS)	1.75
FTL0457	FTT0391c	Stress	Cold shock protein similar to CspA or CspB	1.69
FTL1367	FTT0746c	Stress	Conserved hypothetical membrane protein; fusaric acid resistance	1.66
FTL0859	FTT0595c	Metal	Rubredoxin with nonheme iron-binding domain	1.68
FTL0722	FTT1222	IM	DedA family protein; membrane homeostasis	1.62
FTL1835	FTT0026c	Metal	FslD; conserved drug resistance transporter Bcr/CflA subfamily	1.61
FTL0586	FTT1528	IM	FadD-like long chain fatty acid CoA ligase	1.58
FTL0547	FTT1561	LPS/Capsule	KdtA; Glycosyltransferase	1.56
FTL0723	FTT1221	Metal	BolA-like protein; iron regulation	1.53
FTL0592	FTT1464c	LPS/Capsule	WbtA; dTDP-glucose 46-dehydratase capsule biosynthesis protein	1.47
FTL1231	FTT0970	Metal	conserved hypothetical protein; FeS assembly transcriptional regulator	1.44
FTL1372	FTT0742	OMP	Hypothetical lipoprotein	-1.76
FTL1868	FTT1727c	IM	Major Facilitator Superfamily (MFS) Bcr/CflA subfamily	-1.65
FTL0319	FTT0827c	IM	Hypothetical protein yieG; Xanthine/uracil/vitamin C permease;	-1.59
FTL0192	FTT0282	Metal	Heme/copper-type cytochrome/quinol oxidase	-1.53
FTL0765	FTT1591	OMP	VacJ lipoprotein	-1.46
FTL1882	FTT1738c	IM	Potassium-transporting ATPase B chain	-1.45
FTL0393	FTT0891	LPS/Capsule	LpxE lipid 1' phosphatase	-1.40
FTL1420	FTT0801c	LPS/Capsule	Carbohydrate/purine kinase pfkB family protein	-1.39
FTL1432	FTT0789	LPS/Capsule	D-ribulose-phosphate 3-epimerase	-1.36
FTL0473	FTT0403	Metal	Peptide deformylase; Fe(II) metalloenzyme	-1.33

^a^ Gene loci were classified based on predicted functions (*e*.*g*. metal homeostasis, stress response, or LPS/capsule synthesis) or putative subcellular localization (inner membrane–IM; outer membrane–OM).

### Δ*fmvB* is attenuated in a mouse infection model

**Fig 7:**
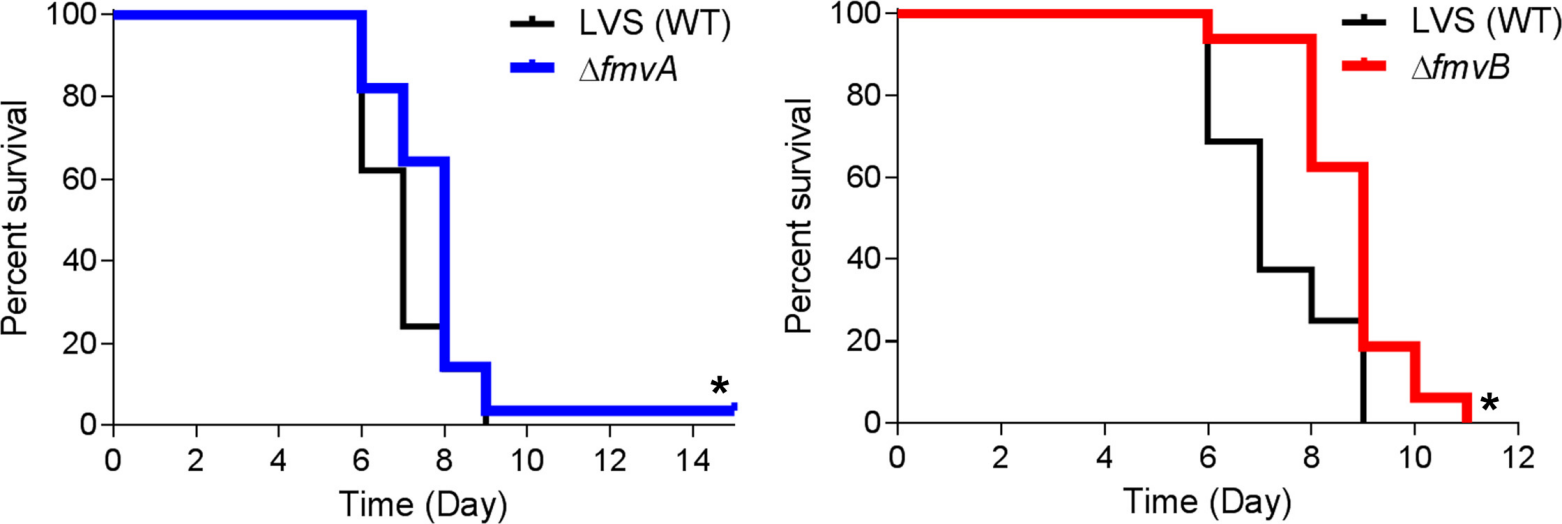
Δ*fmvB* is attenuated in a mouse pulmonary infection model. Female C3H/HeN mice were intranasally-infected with either wild-type (WT) LVS (n = 26, 13,340 CFU), Δ*fmvA* (n = 25, 13,040 CFU), or wild-type (WT) LVS (n = 16, 15,200 CFU), Δ*fmvB* (n = 16, 16,950 CFU). Survival was monitored until all mice succumbed to infection or through day 15 post-infection. Significant differences in delayed time-to-death are indicated by * (*P* < 0.05). Two independent experiments of similar design were performed to confirm reproducibility.

As noted above, increased expression of *fmvA* and *fmvB* in infected mice (Fig 2 and Fig B in S1 File) indicated that these two genes may be important for *F*. *tularensis* virulence. To test this, we intranasally-infected mice with approximately 10^4^ CFU of either wild-type LVS, Δ*fmvA*, or Δ*fmvB* and monitored animal health for 15 days. Whereas all wild-type-infected mice succumbed to infection within 9 days (mean time-to-death 7 days; Fig 7), Δ*fmvA*-infected mice exhibited a 1-day delayed time-to-death (*P* < 0.05; Fig 7), and Δ*fmvB*-infected mice exhibited a 2-day delayed time-to-death (*P* < 0.05; Fig 7). In follow-up studies, the attenuation of Δ*fmvB* was more closely examined by reducing the infectious doses of both wild-type LVS and Δ*fmvB* to approximately 6 × 10^3^ CFU. When the infectious doses were reduced, Δ*fmvB* demonstrated a 5-day delayed time-to-death compared to wild-type LVS (*P* < 0.05; Fig H in S1 File). Importantly, despite this reduced infectious dose, all wild-type-infected mice still succumbed to infection within 9 days.

**Fig 8:**
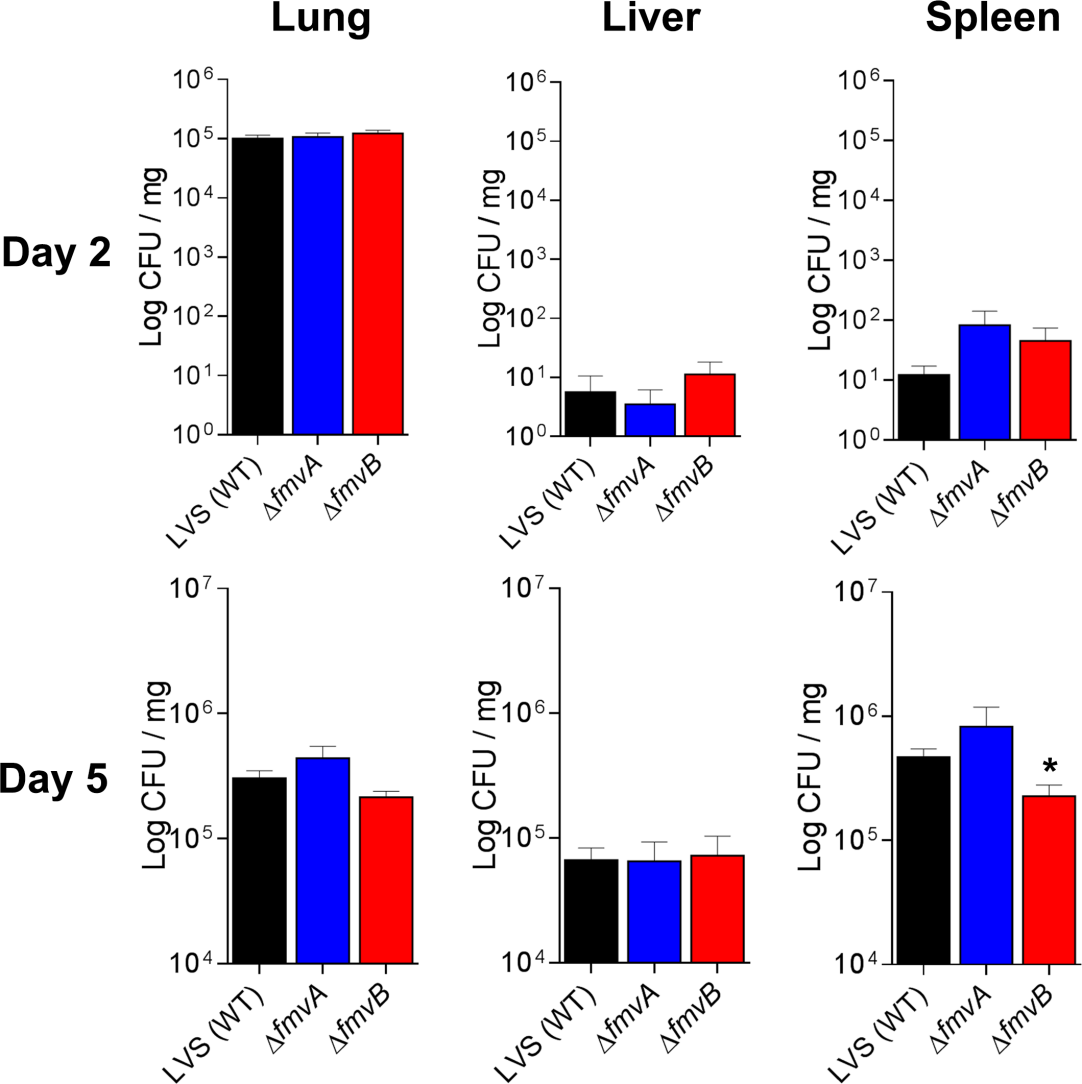
Δ*fmvB*-infected mice have significantly reduced bacterial burdens in their spleens on day 5 post-infection. Groups of 8 female C3H/HeN mice were intranasally-infected with 10^4^ CFU of either wild-type (WT) LVS (13,350 CFU), Δ*fmvA* (15,850 CFU), or Δ*fmvB* (14,200 CFU). On days 2 and day 5 post-infection, lungs, livers, and spleens were harvested from 4 mice per group and plated to enumerate bacterial burdens (CFU per mg of tissue). Significant differences, compared to WT, are indicated by * (*P* < 0.05). Two independent experiments of similar design were performed to confirm reproducibility.

Given the above results, we reasoned that delayed time-to-death for Δ*fmvA-* or Δ*fmvB-* infected mice could be due to two major factors: (1) reduced mutant replication *in vivo* [compared with wild-type]; and/or (2) differences in immune responses to wild-type and mutant bacteria [*i*.*e*. increased LPS expression by Δ*fmvB* may enhance immunogenicity]. To test the first possibility, lungs, livers, and spleens were harvested from mice infected with wild-type LVS, Δ*fmvA*, or Δ*fmvB* and tissues were homogenized and plated to enumerate bacterial numbers on days 2 and 5 post-infection. On day 2 post-infection, no significant differences in lung, liver, or spleen bacterial burdens were observed for wild-type LVS-, Δ*fmvA-*, or Δ*fmvB-*infected tissues (Fig 8). However, on day 5 post-infection, Δ*fmvB*-infected mice had significantly lower numbers of bacteria in their spleens (Fig 8). Given the observed delayed time-to-death for Δ*fmvB*-infected mice (Fig 7 and Fig H in S1 File), it is possible that lower numbers of bacteria in the spleens of Δ*fmvB*-infected mice on day 5 post-infection (Fig 8) accounted for the delayed time-to-death. To more closely examine potential replication defects of Δ*fmvA* or Δ*fmvB* in different cell types, we performed *in vitro* infection studies using bone marrow-derived mouse macrophages and the hepatocyte cell line HepG2. However, Δ*fmvA* and Δ*fmvB* were found to replicate similarly as wild-type LVS in both cell types (Fig I in S1 File).

**Fig 9:**
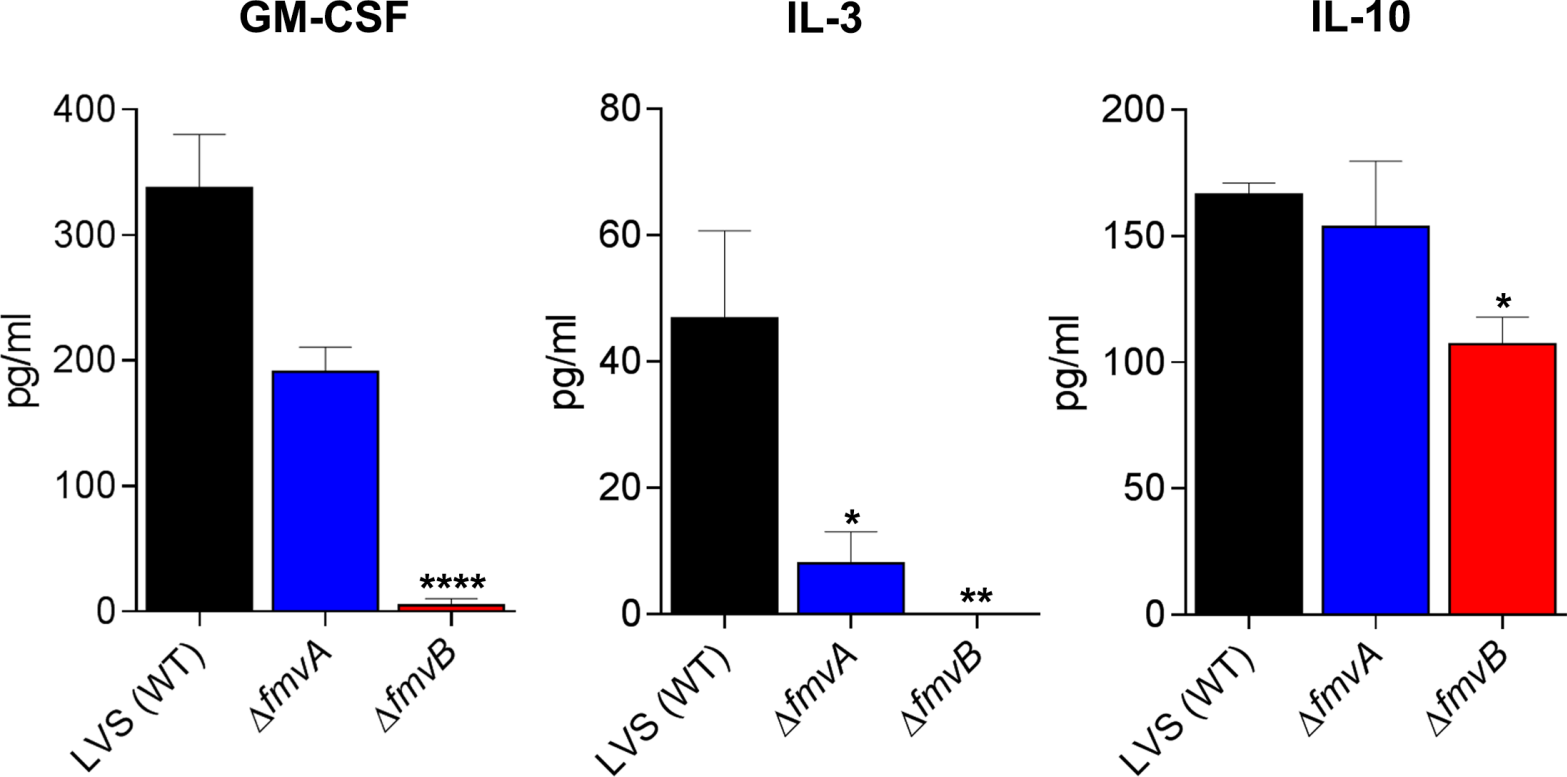
Reduced cytokine expression in Δ*fmvB*-infected spleens on day 5 post-infection. Groups of 8 female C3H/HeN mice were intranasally-infected with 10^4^ CFU of either WT (WT) LVS (18,850 CFU), Δ*fmvA* (19,750 CFU), or Δ*fmvB* (17,500 CFU). On days 2 and day 5 post-infection, lungs, livers, and spleens were harvested from 4 mice per group, tissues were homogenized, and 23 cytokines were quantitated from each tissue by multiplex cytokine analysis. Significant differences, compared to WT, are indicated by * (*P* < 0.05), ** (*P* < 0.01), or **** (*P* < 0.0001). Two independent experiments of similar design were performed to confirm reproducibility.

To test the possibility that wild-type LVS, Δ*fmvA*, and Δ*fmvB* stimulate different immune responses that correlate with delayed time-to-death or survival from infection, we examined the levels of 23 different cytokines from tissue lysates of infected animals on days 2 and 5 post-infection. Whereas no significant differences in cytokine expression levels were observed in any tissues on day 2 post-infection (data not shown), Δ*fmvB*-infected mice produced significantly less GM-CSF, IL-3, and IL-10 in their spleens on day 5 post-infection, compared with wild-type infected mice (Fig 9). Interestingly, this reduced cytokine expression by Δ*fmvB* on day 5 post-infection (Fig 9) correlated with reduced bacterial numbers in the spleens of Δ*fmvB*-infected mice on day 5 post-infection (Fig 8). Δ*fmvA*-infected mice also produced less GM-CSF and IL-3 in their spleens, compared with wild-type infected mice (Fig 9), but only IL-3 expression was significantly lower in the spleens of Δ*fmvA*-infected mice. Given that GM-CSF and IL-3 are part of the ‘cytokine storm’ that correlates with infection-related disease severity and death [69, 70], these data indicate that lower levels of GM-CSF and IL-3 may have contributed to the delayed time-to-death observed following infection with either Δ*fmvA* or Δ*fmvB*.

## Discussion

When the SchuS4 genome was published in 2005 [21], the authors noted the presence of five orthologs of unknown function and these orthologs had no homologs in other bacteria. Three of these genes, *fslE*, *fupA*, and *fupB*, have been extensively studied and shown to play important roles in iron acquisition in *F*. *tularensis* [23–26, 28]. Prior to this study, FmvA and FmvB had not been characterized. Despite that fact that FmvA and FmvB share 37% to 54% identity to those iron acquisition proteins (Fig 1 and Table B in S1 File), we showed here that FmvA and FmvB do not appear to be involved in iron acquisition. Because of the complex nature of iron uptake by *F*. *tularensis*, including functional compensation among the three previously-characterized paralogs [23, 26], our studies required the use of single and double-gene deletion mutants, growth studies in several different iron-limiting media, and transcriptional analyses to confirm that *fmvA* and *fmvB* were not involved in *F*. *tularensis* iron uptake. Overall, our findings highlighted the limitations of using sequence homology to predict protein function. Similar limitations in predicting protein function have been noted by other groups, including a report that despite 41% amino acid identity to the *Moraxella catarrhalis* heme receptor HumA, the *Neisseria meningitidis* protein ZnuD actually imports zinc [71]. Other studies have reported that bacterial metal transporters can indiscriminately bind and transport a wide array of cations [12–14, 57, 58]. Particularly relevant to this study, the *E*. *coli* and *Salmonella typhimurium* CorA magnesium transport proteins also have been shown to transport cations such as manganese (Mn^2+^), copper (Co^2+^), and nickel (Ni^2+^) [72, 73]. As such, we next examined the possibility that FmvA or FmvB could transport cations other than iron, including magnesium or calcium. Using a variety of approaches, we demonstrated that FmvB was outer membrane localized (Fig 4), responsive to magnesium (*i*.*e*. *fmvB* expression increased in low magnesium; Fig 3), and plays a role in LPS maintenance (Fig 5). We were not able to demonstrate that FmvB was required for growth in magnesium limitation (Figs E and F in S1 File), but given the above noted functional redundancy of iron import proteins and the upregulation of at least four putative metal importers in Δ*fmvB* (e.g. FTL1199, FTL1833, FTL0859, and FTL1835; Table 2), it is possible that other proteins compensated for the loss of FmvB. Despite our best attempts to characterize both FmvA and FmvB, we still do not know the function of FmvA and only can speculate about the function of FmvB. Importantly, our data do suggest that as an outer membrane-localized protein that is magnesium responsive, FmvB may import magnesium and may interact with LPS assembly proteins to ‘load’ magnesium between adjacent LPS molecules, which is required to negate negative charges and maintain LPS integrity.

Studies to directly test if FmvB is involved in magnesium uptake likely would require the use of radiolabeled magnesium [74], a siderophore-mediated uptake assay [23, 28] and/or purified recombinant proteins [75]. At this time, we do not know if the *Francisella* siderophore [23] is involved in magnesium uptake. In addition, despite our previous studies expressing and purifying recombinant *F*. *tularensis* OMPs from *E*. *coli* [36], we were unable to express and purify sufficient amounts of recombinant FmvB for these types of studies (data not shown). Another possibility includes feeding mice a low magnesium diet [76] and then infecting those mice with Δ*fmvB* but potential functional redundancy (noted above) likely would complicate these experiments. Finally, it is worth noting that magnesium studies are particularly challenging because magnesium is required for many processes associated with bacterial growth and virulence. First, relative to other cations, magnesium is stored at high concentrations (0.5 to 1 mM) in the bacterial cytoplasm [77, 78]. Second, our speculation that other proteins may import magnesium in the absence of FmvB is supported by the fact that *Salmonella enterica* serovar *Typhimurium* encodes at least three known magnesium transporters [79]. Finally, as noted above, high levels of magnesium are required to maintain LPS integrity in the outer membrane [16]. Taken together, our studies linking *F*. *tularensis* FmvB and magnesium have revealed an entirely new area of research in the *Francisella* field and provide additional information to the bacterial pathogenesis and magnesium transport fields.

This study demonstrated the power of constantly-improving bioinformatic prediction programs to predict the localization and secondary structure of FmvA and FmvB. Whereas PSORTb has been reported to be the most precise bacterial subcellular localization (SCL) prediction program available [36, 44], we also used a series of six beta barrel prediction programs to analyze FmvA and FmvB, given that beta-barrel proteins are found exclusively in the outer membrane of Gram-negative bacteria [43, 45]. Taken together, six of seven prediction programs (PSORTb and the six beta barrel prediction programs) indicated that FmvA and FmvB were outer membrane beta barrel proteins (Table 1 and Fig A in S1 File). To confirm these predictions, we used spheroplasting, osmotic lysis, and sucrose density gradient centrifugation to prove that FmvB was outer membrane (OM)-localized. OM-localization studies only were performed for FmvB, due to the upregulation of *fmvB* in magnesium-limiting medium (Fig 3), increased LPS expression in Δ*fmvB* (Fig 5), and delayed time-to-death of Δ*fmvB*-infected mice (Fig 7 and Fig H in S1 File). In addition, OM-localization of FmvB only was possible following the addition of a 6x histidine fusion tag to the C-terminal end of FmvB using a genetic complementation construct. We attempted multiple approaches to generate polyclonal monospecific antibodies against FmvB, including the use of recombinant protein immunization, protein-specific peptide immunization, and immunization regimens with combinations of recombinant proteins and peptides. However, all of our attempts to generate FmvB-specific antisera failed, likely due to the high degree of amino acid sequence identity among the five paralogs. At this time, we do not know the subcellular localization of FmvA but, given the strength of the bioinformatic predictions, we are fairly confident that FmvA is an outer membrane beta barrel.

Because metals are tightly-sequestered by the host [17, 18], pathogenic bacteria encode sophisticated mechanisms to capture or extract metals *in vivo* [12, 14]. Whereas most metal studies have focused on iron uptake by various pathogenic bacteria, it is now well-appreciated that metals such as magnesium and calcium also are important for bacterial virulence, can serve as coenzymes for many bacterial processes, protect against free radical damage, are important to maintain membrane integrity, and can act as second message molecules [12, 14, 15]. As noted throughout this study, *F*. *tularensis* iron-uptake systems have been well characterized and are known to be required for *F*. *tularensis* growth and virulence [23, 26, 28, 35, 80]. As such, we initially aimed to test the hypothesis that *F*. *tularensis* upregulates known iron-acquisition genes *in vivo*. Using similar logic, we speculated that FmvA and FmvB, because of their homology to known iron-acquisition genes, also would be upregulated *in vivo*, and that *in vivo* upregulation would correlate with iron/metal uptake and/or virulence. These hypotheses were based on other studies that used *in vivo* transcriptional analyses to identify novel virulence factors in other pathogenic bacteria [54–56]. Indeed, our *in vivo* transcriptional analysis demonstrated that *fslE*, one of the major *F*. *tularensis* iron acquisition genes [25], was dramatically upregulated in SchuS4-infected mice (up to 74-fold; Fig 2), highlighting that iron is extremely limiting *in vivo*. Interestingly, our analysis of *fslE* expression in various metal-depleted conditions found that *fslE* was upregulated not only in response to iron limitation, but also to calcium and magnesium limitation (up to 40-fold; Fig 3). Thus, although we did not examine all possible metal-depleting conditions, our data indicate that *fslE* does not specifically respond to iron-limitation but, instead, may broadly respond to or be regulated by many different metal-limiting conditions. Despite our discovery of this non-specific metal responsive mechanism in *F*. *tularensis*, it is unlikely that *in vivo* upregulation of *fslE* correlates with *F*. *tularensis* virulence, as previous studies have demonstrated that *fslE* is not required for LVS or SchuS4 virulence [23, 26]. The second test of our *in vivo* upregulation hypothesis was applied to *fupA*, which previously was shown to be required for *F*. *tularensis* iron acquisition [23] and virulence [22, 23]. SchuS4 *fupA* expression was virtually unchanged *in vivo* (Fig 2) and LVS *fupAB* expression was unchanged in various metal limiting conditions (Fig 3), suggesting that SchuS4 *fupA* and LVS *fupAB* are constitutively expressed and that not all *F*. *tularensis* virulence factors are upregulated *in vivo*. Finally, our hypotheses were not necessarily true for *fmvA* or *fmvB*. For *fmvA*, gene expression was virtually unchanged *in vivo* (Fig 2) or in metal-depleted media (Fig 3) and Δ*fmvA* did not result in substantial attenuation (Fig 7). As noted above, we did not examine all possible metal-depleting conditions, so it remains possible that FmvA may be involved in the uptake of metals not tested here. For *fmvB*, gene expression was significantly upregulated both *in vivo* (Fig 2) and in magnesium-depleted media (Fig 3) and we found that Δ*fmvB* was significantly attenuated in mice (Fig 7 and Fig H in S1 File). Thus, of the paralogs examined in this study, only FmvB satisfies the criteria of being upregulated *in vivo*, likely playing a role in metal uptake, and playing a role in virulence. Other investigators have faced similar hurdles when using transcriptional analyses to identify *F*. *tularensis* virulence genes. For example, DNA microarray analysis of *F*. *tularensis* SchuS4 from primary mouse bone marrow derived macrophages revealed 152 upregulated genes. Of those, the investigators generated 10 isogenic mutants, infected macrophages with individual mutants, and found that only 3 of 10 were attenuated [81]. In summary, additional studies are needed to further test this hypothesis and fully understand the importance of *in vivo* upregulated genes and *F*. *tularensis* virulence.

Due to our findings that FmvB was outer membrane-localized (Fig 4) and upregulated in magnesium limitation (Fig 3), we suspected that LPS expression might be altered in *ΔfmvB* given that magnesium is required to neutralize LPS negative charges and many bacteria are known to alter their LPS in magnesium limitation [16]. Indeed, both immunoblot and flow cytometry analyses demonstrated that Δ*fmvB* expressed 50% more LPS than wild-type *F*. *tularensis* in magnesium-limiting conditions (Fig 5). At this time, it is difficult to definitively explain why Δ*fmvB* expresses more LPS than wild-type. However, our RNAseq analysis revealed that two LPS synthesis genes, *kdtA* and *wbtA* were increased approximately 50% in Δ*fmvB* (Table 2). *kdtA* encodes a putative core oligosaccharide KDO transferase and *wbtA* has been associated with O-antigen and capsule production in *F*. *tularensis* [63, 82]. Conversely, RNAseq analysis also indicated that three LPS/capsule-associated genes were downregulated approximately 40% in Δ*fmvB* (Table 2), including *lpxE* (lipid 1’ phosphatase; [83]), FTL1420 (carbohydrate kinase family protein), and FTL1432 (D-ribulose-phosphate 3-epimerase; [84]). Finally, it remains possible that the gene expression changes noted above are not solely due to loss of *fmvB* but may have been confounded by low magnesium levels in the growth medium. However, wild-type LVS also was grown in low magnesium and analyzed by RNAseq analysis, so we have discounted this possibility.

Because flow cytometry analysis demonstrated that increased LPS was surface localized, we examined Δ*fmvB* bacterial morphology by electron microscopy (EM), finding that Δ*fmvB* bacteria possessed increased numbers of protrusions, compared with wild-type *F*. *tularensis* (Fig 6). At this time, we do not know the relevance or function of these protrusions but it is possible that these are outer membrane vesicles (OMVs). Similar surface protrusions/outer membrane vesicles have been reported in *F*. *tularensis* and it is well known that Gram-negative bacteria produce outer membrane vesicles for multiple biological processes, including for virulence factor secretion or for quorum sensing [85, 86]. Based on our EM data, we speculated that Δ*fmvB* may be partially deficient in releasing OMVs from the bacterial surface. If FmvB does play a role in OMV release, this could explain why more LPS was retained on Δ*fmvB* bacteria. Reduced OMV release also may help explain the observed attenuation (Fig 7) and reduced cytokine stimulation (Fig 8) of Δ*fmvB* in mice. However, additional studies are needed to confirm the role of FmvB in OMVs and increased LPS on the bacterial surface.

We observed significant attenuation of Δ*fmvB* in pulmonary mouse infections (Fig 7 and Fig H in S1 File) and reduced levels of GM-CSF, IL-3, and IL-10 in the spleens of Δ*fmvB*-infected mice on day 5 post-infection (Fig 9), indicating that FmvB is involved in *F*. *tularensis* virulence. In addition, Δ*fmvB* was deficient for bacterial dissemination to and replication in spleens on day 5 post-infection (Fig 8). It is possible that significantly reduced bacterial burdens in mouse spleens on day 5 correlated with the delayed time-to-death observed for Δ*fmvB*. It also is possible that significantly reduced levels of GM-CSF, IL-3, and IL-10 in the spleens of Δ*fmvB*-infected mice on day 5 post-infection correlated with the observed delayed time-to-death. IL-3 has been previously shown to induce sepsis in mice [69] and it is well-documented that *F*. *tularensis*-infected animals eventually die from a cytokine storm [87]. GM-CSF also has been implicated in cytokine-induced sepsis and death [70]. Taken together, our findings indicate that Δ*fmvB* attenuation could be due to the reduced ability of Δ*fmvB* to stimulate IL-3 and GM-CSF or delayed IL-3 and GM-CSF production by Δ*fmvB-*infected mice. We also found that Δ*fmvB-*infected mice produced significantly lower levels of IL-10, compared with wild-type infected mice, however these findings are difficult to interpret at this time. Although IL-10 generally has been considered to be an immunosuppressive cytokine [88], IL-10 also has been demonstrated to play important immune regulatory functions during *F*. *tularensis* infection [30]. Given the reduced levels of GM-CSF and IL-3 in Δ*fmvB-*infected mice, lower levels of IL-10 may be needed to reduce the cytokine storm severity.

In conclusion, despite the amino acid identity of FmvA and FmvB to the previously-studied *F*. *tularensis* iron acquisition genes FslE, FupA, and FupB, neither FmvA nor FmvB are involved in iron acquisition. Through a series of gene expression analyses in metal-depleted media, outer membrane localization studies, and LPS expression analyses, our results indicate that FmvB may play a role in magnesium transport and LPS maintenance. Additionally, Δ*fmvB* was observed to possess increased numbers of surface protrusions, compared with wild-type *F*. *tularensis*, suggesting that FmvB may play a role in OMV release. Finally, Δ*fmvB* was found to be significantly attenuated in a pulmonary mouse infection model, highlighting its role in *F*. *tularensis* virulence. This is the first study to characterize and examine the functions of these iron-acquisition paralogs.

## Supporting Information

S1 File **Table A. Oligonucleotide primers used for qRT-PCR. Table B. FmvA and FmvB are paralogs of known iron acquisition genes. Table C. Δ*fmvB* does not have increased sensitivity to antimicrobial agents. Fig A. PRED-TMBB modeling of FmvA and FmvB.** (A) Two-dimensional topology models for FmvA and FmvB were predicted using PRED-TMBB and the results were viewed using TMRPres2D. (B) Three-dimensional models for FmvA and FmvB were predicted by TMBpro and viewed using Swiss-PdbViewer. **Fig B. Gene expression analysis from *F*. *tularensis* LVS-infected mice.** Groups of 4 female C3H/HeN mice were intranasally-infected with 6925 CFU of *F*. *tularensis* strain LVS. Lungs, livers, and spleens were harvested on days 2 and day 5 post-infection. qRT-PCR was performed to quantitate the relative gene expression of *fslE*, *fupAB*, *fmvA*, and *fmvB* during *in vivo* infection compared to LVS grown in laboratory medium. Data were normalized to ‘housekeeping’ gene DNA gyrase α subunit (*gyrA*; FTL0533). Significant differences are indicated by * (*P* < 0.05). **Fig C. Δ*fmvA* and Δ*fmvB* do not exhibit growth defects in iron-limitation.** Wild-type (WT) LVS, Δ*fmvA*, and Δ*fmvB* were cultured in either iron replete or iron-limiting chemically defined medium (CDM). Δ*fupAB* served as a positive control for growth defects in iron-limiting medium. Indicated bacterial strains were grown in either: (A) Iron replete liquid CDM; (B) Iron-limiting liquid CDM; (C) Iron replete CDM agar; or (D) Iron-limiting CDM agar. Two independent experiments of similar design were performed to confirm reproducibility. Representative results shown. **Fig D. Δ*fmvA*/Δ*fupAB* and Δ*fmvB*/Δ*fupAB* double mutants do not exhibit growth defects in iron-limiting medium.** Wild-type (WT) LVS, Δ*fmvA*/Δ*fupAB*, and Δ*fmvB*/Δ*fupAB* were cultured in either iron replete or iron-limiting CDM. Δ*fupAB* served as a positive control for growth defects in iron-limiting medium. Indicated bacterial strains were grown in either: (A) Iron replete liquid CDM; (B) Iron-limiting liquid CDM; (C) Iron replete CDM agar; or (D) Iron-limiting CDM agar. Two independent experiments of similar design were performed to confirm reproducibility. Representative results shown. **Fig E. Δ*fmvA* and Δ*fmvB* do not exhibit growth defects in magnesium-limiting medium.** Wild-type (WT) LVS, Δ*fmvA*, and Δ*fmvB* were cultured in either magnesium replete or magnesium-limiting CDM. Indicated bacterial strains were grown in either: (A) Magnesium replete liquid CDM or (B) Magnesium-limiting liquid CDM. Two independent experiments of similar design were performed to confirm reproducibility. Representative results shown. **Fig F. Δ*fmvB*/Δ*fupAB* and Δ*fmvB*/Δ*fslE* double mutants do not exhibit growth defects in magnesium-limitation.** (A) and (B) Wild-type (WT) LVS, Δ*fmvB*, Δ*fslE*, and Δ*fmvB/*Δ*fslE* were cultured in either magnesium replete or magnesium-limiting liquid CDM, with Δ*fslE* serving as a control. (C) and (D) WT LVS, Δ*fmvB*, Δ*fupAB*, and Δ*fmvB*Δ*fupAB* were cultured in either magnesium replete or magnesium-limiting liquid CDM, with Δ*fupAB* serving as a control. Two independent experiments of similar design were performed to confirm reproducibility. Representative results shown. **Fig G. Δ*fmvB* does not have increased sensitivity to antimicrobial peptides.** WT LVS and Δ*fmvB* were incubated in the presence of the indicated antimicrobial peptides for 2 h at 37°C, samples were then serially-diluted in PBS, and plated onto sMHA for bacterial enumeration. Two independent experiments of similar design were performed to confirm reproducibility. Representative results shown. **Fig H. Δ*fmvB* is more attenuated in a mouse pulmonary infection model when the infectious dose is reduced.** Groups of 5 female C3H/HeN mice were intranasally-infected with either wild-type (WT) LVS (6925 CFU) or Δ*fmvB* (5750 CFU). Survival was monitored until all mice succumbed to infection. Significant differences in delayed time-to-death are indicated by * (*P* < 0.05). **Fig I. Neither Δ*fmvA* nor *ΔfmvB* are attenuated *in vitro*.** (A) 1 ☓10^5^ mouse bone marrow derived macrophages were infected at a multiplicity of infection (MOI) of 10 bacteria (wild-type LVS, Δ*fmvA*, or Δ*fmvB*) per cell (10:1). (B) 1 ☓10^5^ HepG2 cells were infected at a MOI of 50:1. Following either 2 h or 16 h of infection, cells were lysed, serially-diluted in PBS, and plated onto sMHA for bacterial enumeration. Two independent experiments of similar design were performed to confirm reproducibility.
(PDF)

S2 File Data file. **Table D. Complete data from RNA sequencing analysis of wild-type LVS and ∆fmvB**.
(XLSX)
