# Supplementary material for: FmvB: A Francisella tularensis Magnesium-Responsive Outer Membrane Protein that Plays a Role in Virulence
Source: PLoS One. 2016 Aug 11;11(8):e0160977. doi: 10.1371/journal.pone.0160977 (PMC4981453; doi:10.1371/journal.pone.0160977)
Supplement: S1 File — Table A. Oligonucleotide primers used for qRT-PCR. Table B. FmvA and FmvB are paralogs of known iron acquisition genes. Table C. ΔfmvB does not have increased sensitivity to antimicrobial agents. Fig A. PRED-TMBB modeling of FmvA and FmvB. (A) Two-dimensional topology models for FmvA and FmvB were predicted using PRED-TMBB and the results were viewed using TMRPres2D. (B) Three-dimensional models for FmvA and FmvB were predicted by TMBpro and viewed using Swiss-PdbViewer. Fig B. Gene expression analysis from F. tularensis LVS-infected mice. Groups of 4 female C3H/HeN mice were intranasally-infected with 6925 CFU of F. tularensis strain LVS. Lungs, livers, and spleens were harvested on days 2 and day 5 post-infection. qRT-PCR was performed to quantitate the relative gene expression of fslE, fupAB, fmvA, and fmvB during in vivo infection compared to LVS grown in laboratory medium. Data were normalized to ‘housekeeping’ gene DNA gyrase α subunit (gyrA; FTL0533). Significant differences are indicated by * (P < 0.05). Fig C. ΔfmvA and ΔfmvB do not exhibit growth defects in iron-limitation. Wild-type (WT) LVS, ΔfmvA, and ΔfmvB were cultured in either iron replete or iron-limiting chemically defined medium (CDM). ΔfupAB served as a positive control for growth defects in iron-limiting medium. Indicated bacterial strains were grown in either: (A) Iron replete liquid CDM; (B) Iron-limiting liquid CDM; (C) Iron replete CDM agar; or (D) Iron-limiting CDM agar. Two independent experiments of similar design were performed to confirm reproducibility. Representative results shown. Fig D. ΔfmvA/ΔfupAB and ΔfmvB/ΔfupAB double mutants do not exhibit growth defects in iron-limiting medium. Wild-type (WT) LVS, ΔfmvA/ΔfupAB, and ΔfmvB/ΔfupAB were cultured in either iron replete or iron-limiting CDM. ΔfupAB served as a positive control for growth defects in iron-limiting medium. Indicated bacterial strains were grown in either: (A) Iron replete liquid CDM; (B) Iron-limiting liquid CDM; (C) [file pone.0160977.s001.pdf]

Supporting Information – S1 File

Table A. Oligonucleotide primers used for qRT-PCR

Strain	Locus (gene name)	Primer name	Sequence
SchuS4	FTT0025c (*fslE*)	5'-qfslE(S)	5’-ATTTGTCACTTTACCAGGAGAGAGG-3’
		3'-qfslE(S)	5’-TGCTGGAAGAAACAAACTCTCTGTA-3’
	FTT0918 (*fupA*)	5'-qfupA(S)	5’-TGGGCTAGTACTACGACAAAAGAAG-3’
		3'-qfupA(S)	5’-CTACCACCAAGTATCGCAGAATAGT-3’
	FTT0919 (*fupB*)	5'-qfupB(S)	5’-ACAGTTACCAAGTAACCTTTTTGCC-3’
		3'-qfupB(S)	5’-TGTGCATCAGCTTGAATAAATCCAC-3’
	FTT0267 (*fmvA*)	5'-qfmvA(S)	5’-AGGTGGAGTTTTACAGTTCCAAAGT-3’
		3'-qfmvA(S)	5’-CCGTAAGCTAAACCAAAATACCACG-3’
	FTT0602c (*fmvB*)	5'-qfmvB(S)	5’-GCTGGCTTATTTTATGCCGATAAGT-3’
		3'-qfmvB(S)	5’-TAGGAATGCTAGAGTTTTCAGCACC-3’
	FTT1575c (*gyrA*)	5'-qgyrA(S)	5’-AAAGAGATGAGTCACCTGAAGTTGT-3’
		3'-qgyrA(S)	5’-AGGTCTATTGTCACTAAGAGCAACC-3’
	FTT0350 (*rpoA*)	5'-qrpoA(S)	5'-AGATCAGCCAATAGCTACTTTGACA-3'
		3'-qrpoA(S)	5'-TCGGTTGGTATCGCAGAAAGTATTC-3'
LVS	FTL1836c (*fslE*)	5'-qfslE(L)	5’-ATTTGTCACTTTACCAGGAGAGAGG-3’
		3'-qfslE(L)	5’-TGCTGGAAGAAACAAACTCTCTGTA-3’
	FTL0439 (*fupAB*)	5'-qfupA(L)	5’-ACCAGTATTTGTGTCAGTCGG-3’
		3'-qfupA(L)	5’-TCGCTGCATTGGTAACTCTAAG-3’
	FTL0147 (*fmvA*)	5'-qfmvA(L)	5’-ACATTTGACCAGAACTTATTTAGACG-3’
		3'-qfmvA(L)	5’-GGTTATCTGCCCTTGAGTTGT-3’
	FTL0867c (*fmvB*)	5'-qfmvB(L)	5’-TGGAGCGTGGTATATTGCG-3’
		3'-qfmvB(L)	5’-CATTTTGTAATCGACTTCCGGC-3’
	FTL0533 (*gyrA*)	5'-qgyrA(L)	5’-AAAGAGATGAGTCACCTGAAGTTGT-3’
		3'-qgyrA(L)	5’-AGGTCTATTGTCACTAAGAGCAACC-3’
	FTL1656 (*uvrD*)	5'-quvrD(L)	5'-AGTGAATGTTACGGCAAGGAT-3'
		3'-quvrD(L)	5'-CGTAATGCTTTGATTCTCGCTG-3'

Table B. FmvA and FmvB are paralogs of known iron acquisition genes[a].

	**FslE**	**FupA**	**FupB**	**FmvA**	**FmvB**
**FslE**	100%				
**FupA**	59%	100%			
**FupB**	42%	50%	100%		
**FmvA**	54%	54%	48%	100%	
**FmvB**	49%	48%	37%	47%	100%

[a] BLASTp analysis was performed to compare the amino acid identity of five gene paralogs in *F. tularensis* strain SchuS4. BLASTp analysis was performed by comparing any two of the five amino acid sequences as follows: FslE (FTT0025c), FupA (FTT0918), FupB (FTT0919), FmvA (FTT0267), FmvB (FTT0602c).

Table C. Δ*fmvB* does not have increased sensitivity to antimicrobial agents.

Agent tested	LVS diameter[a]	Δ*fmvB* diameter[a]
Gentamicin	35.8 ±1	36.2 ±2
Ciprofloxacin	58.15 ±1	58.3 ±1
Tetracycline	31 ±0	34 ±1
SDS	28 ±0	28 ±0
Triton X-100	31 ±0	34 ±1
CTAB	15 ±0	14 ±1
Ethidium Bromide	22 ±0	22.15 ±1

[a] Average diameter of the zone of inhibition (including filter disk) in millimeters ± standard error to mean. Diameter of the filter disks were 6.5 mm.

Supporting Information Figures

A.

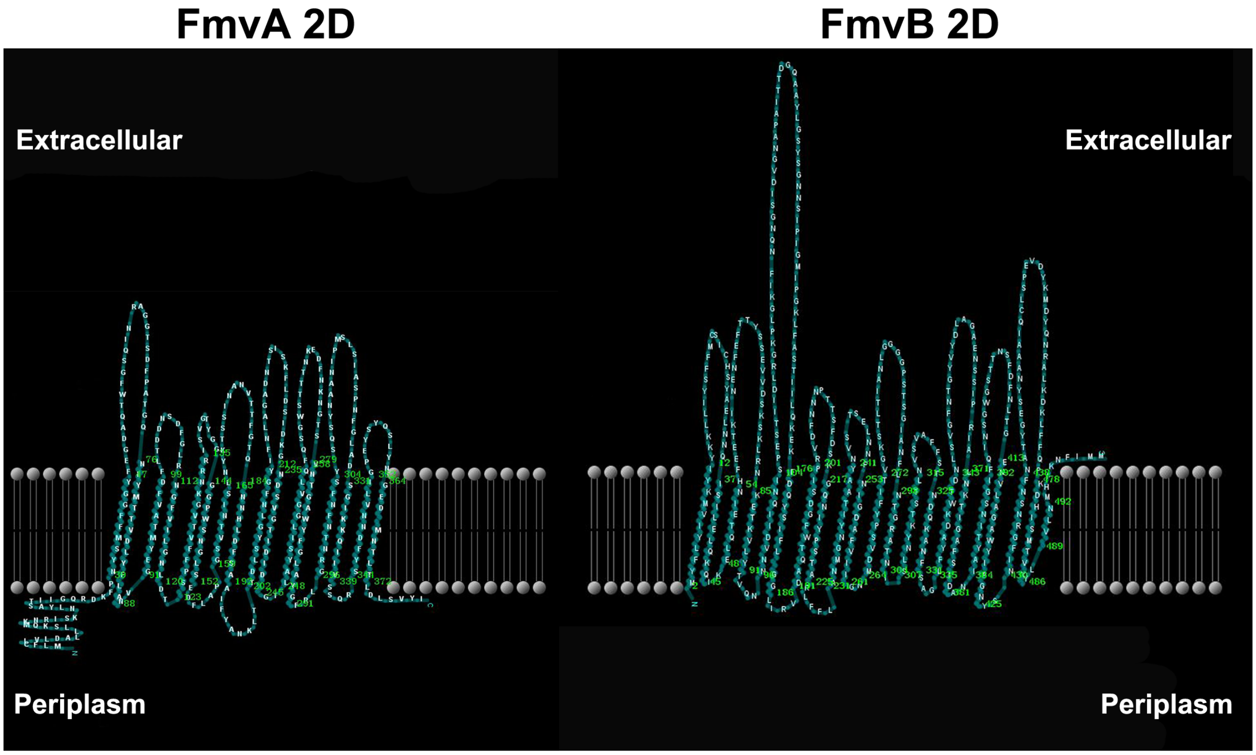

B.

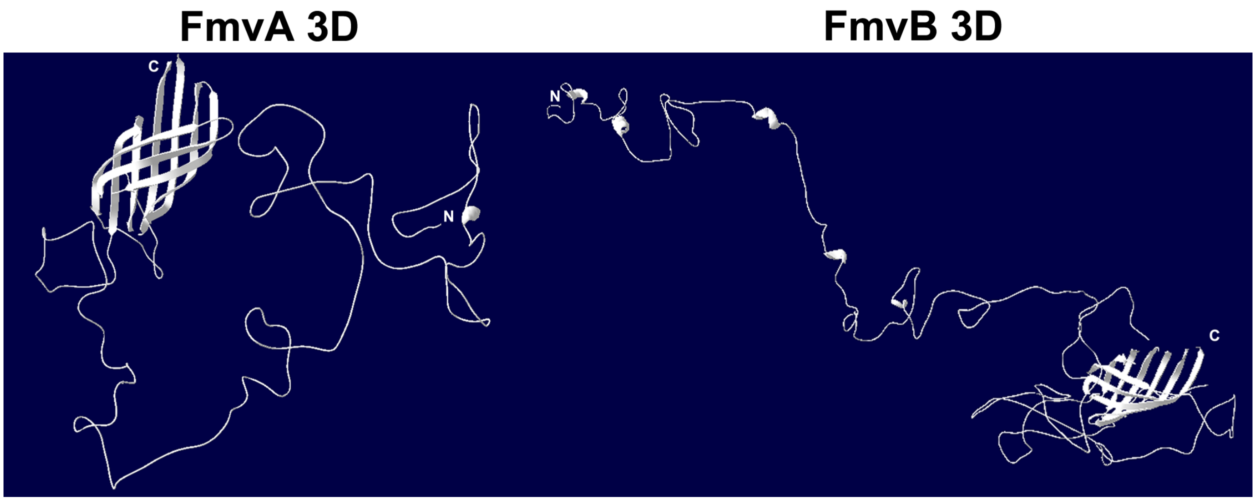

Fig A

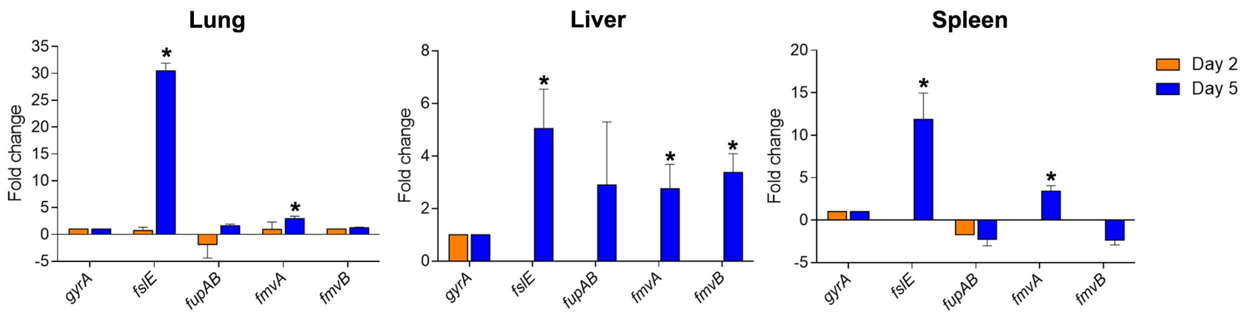
Lung
Liver
Spleen
Fold change
gyrA
fslE
fupAB
fmvA
fmvB
Day 2
Day 5
*

Fig B

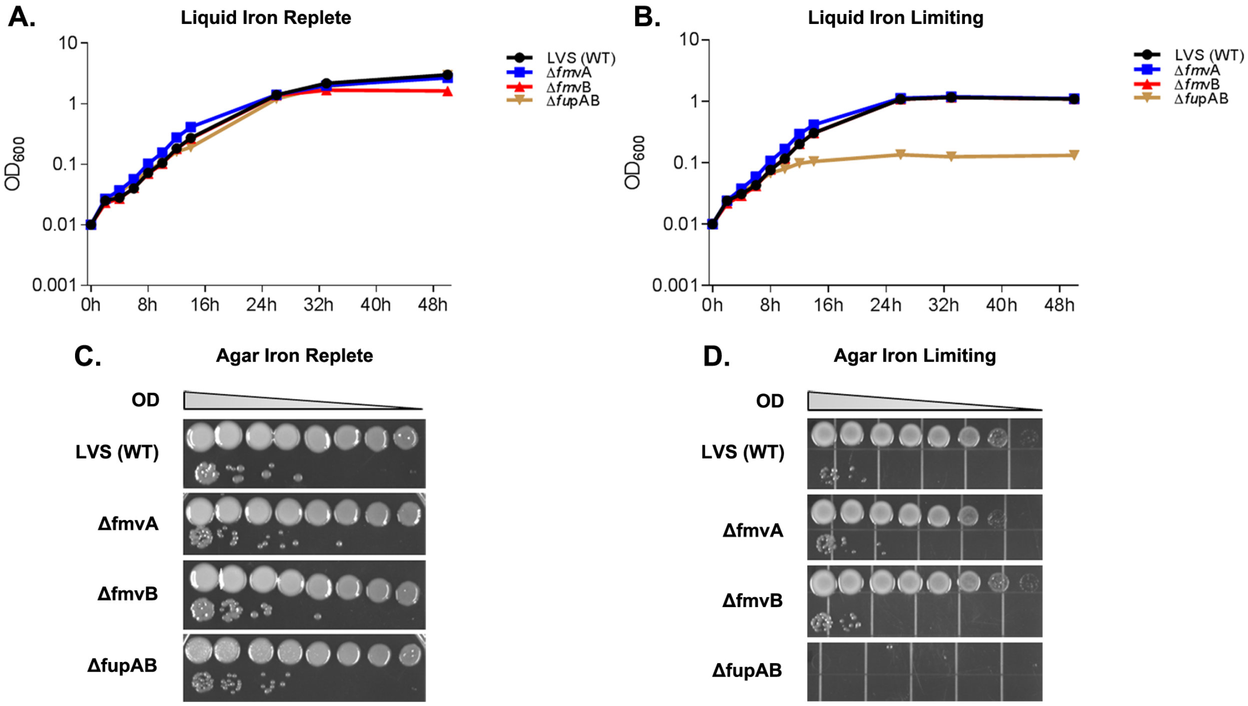

A.
Liquid Iron Replete
10
1
0.1
0.01
0.001
OD600
0h
8h
16h
24h
32h
40h
48h
LVS (WT)
ΔfmvA
ΔfmvB
ΔfupAB
B.
Liquid Iron Limiting
10
1
0.1
0.01
0.001
OD600
0h
8h
16h
24h
32h
40h
48h
LVS (WT)
ΔfmvA
ΔfmvB
ΔfupAB
C.
Agar Iron Replete
OD
LVS (WT)
ΔfmvA
ΔfmvB
ΔfupAB
D.
Agar Iron Limiting
OD
LVS (WT)
ΔfmvA
ΔfmvB
ΔfupAB

Fig C

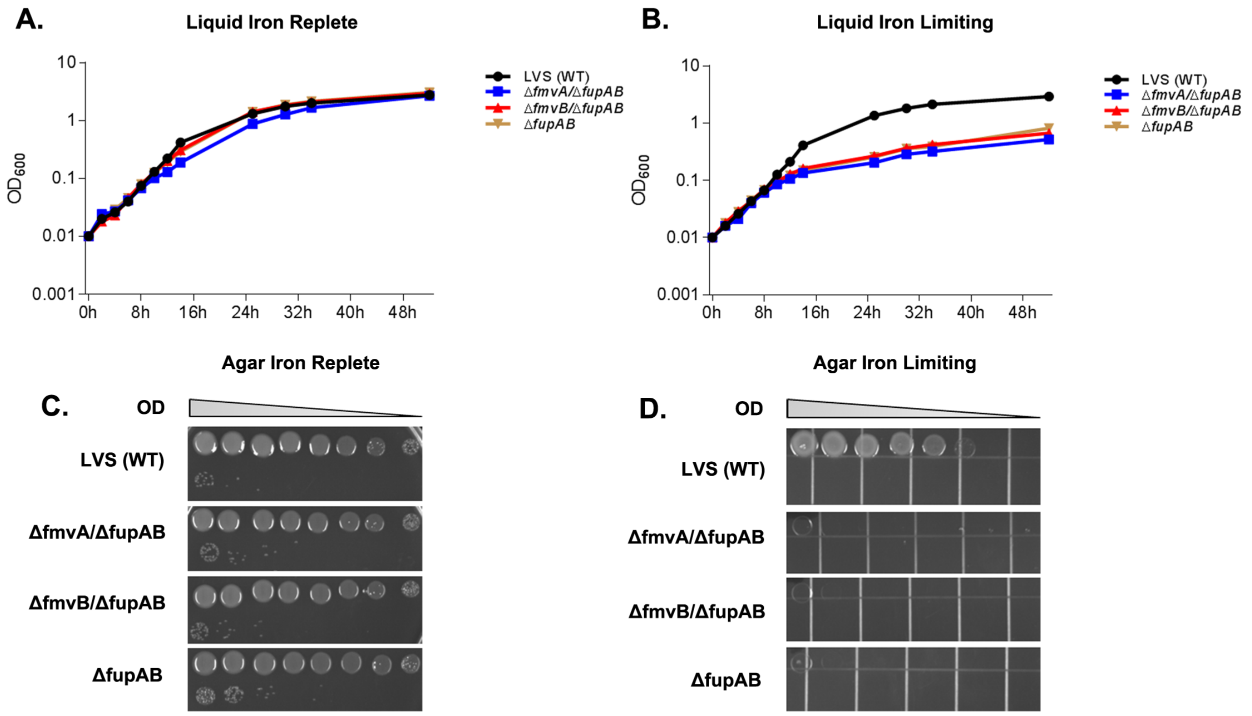
A.
Liquid Iron Replete
B.
Liquid Iron Limiting
OD600
10
1
0.1
0.01
0.001
0h
8h
16h
24h
32h
40h
48h
LVS (WT)
ΔfmvA/ΔfupAB
ΔfmvB/ΔfupAB
ΔfupAB
Agar Iron Replete
Agar Iron Limiting
C.
D.
OD
LVS (WT)
ΔfmvA/ΔfupAB
ΔfmvB/ΔfupAB
ΔfupAB

Fig D

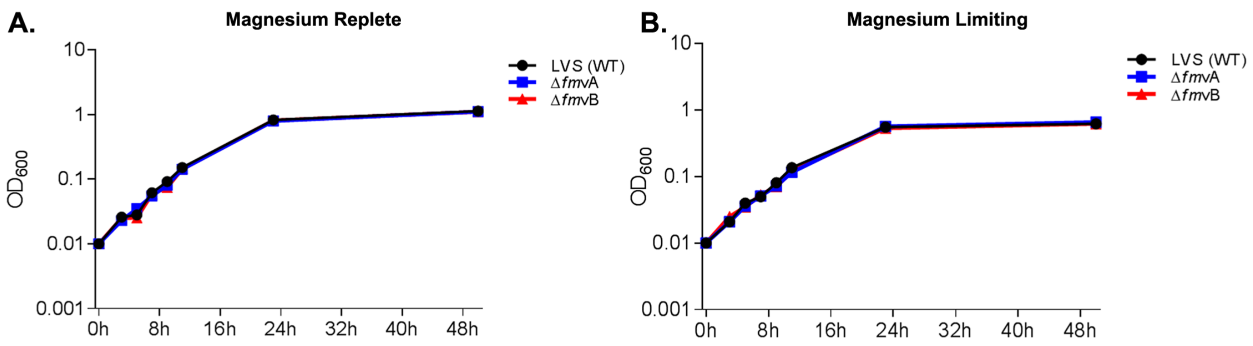

A.
Magnesium Replete
10
1
0.1
0.01
0.001
OD_{600}
0h
8h
16h
24h
32h
40h
48h
LVS (WT)
Δ*fmv*A
Δ*fmv*B
B.
Magnesium Limiting
10
1
0.1
0.01
0.001
OD_{600}
0h
8h
16h
24h
32h
40h
48h
LVS (WT)
Δ*fmv*A
Δ*fmv*B

Fig E

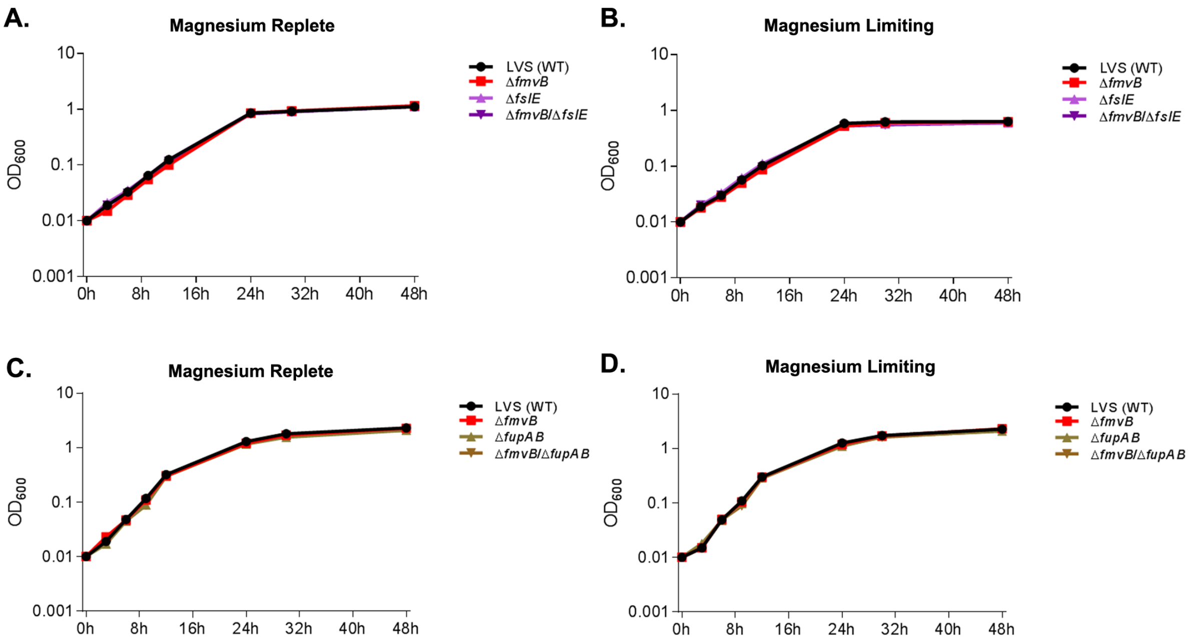
A.
Magnesium Replete
B.
Magnesium Limiting
C.
Magnesium Replete
D.
Magnesium Limiting
OD600
0.001
0.01
0.1
1
10
0h
8h
16h
24h
32h
40h
48h
LVS (WT)
ΔfmvB
ΔfslE
ΔfmvB/ΔfslE
ΔfupAB
ΔfmvB/ΔfupAB

Fig F

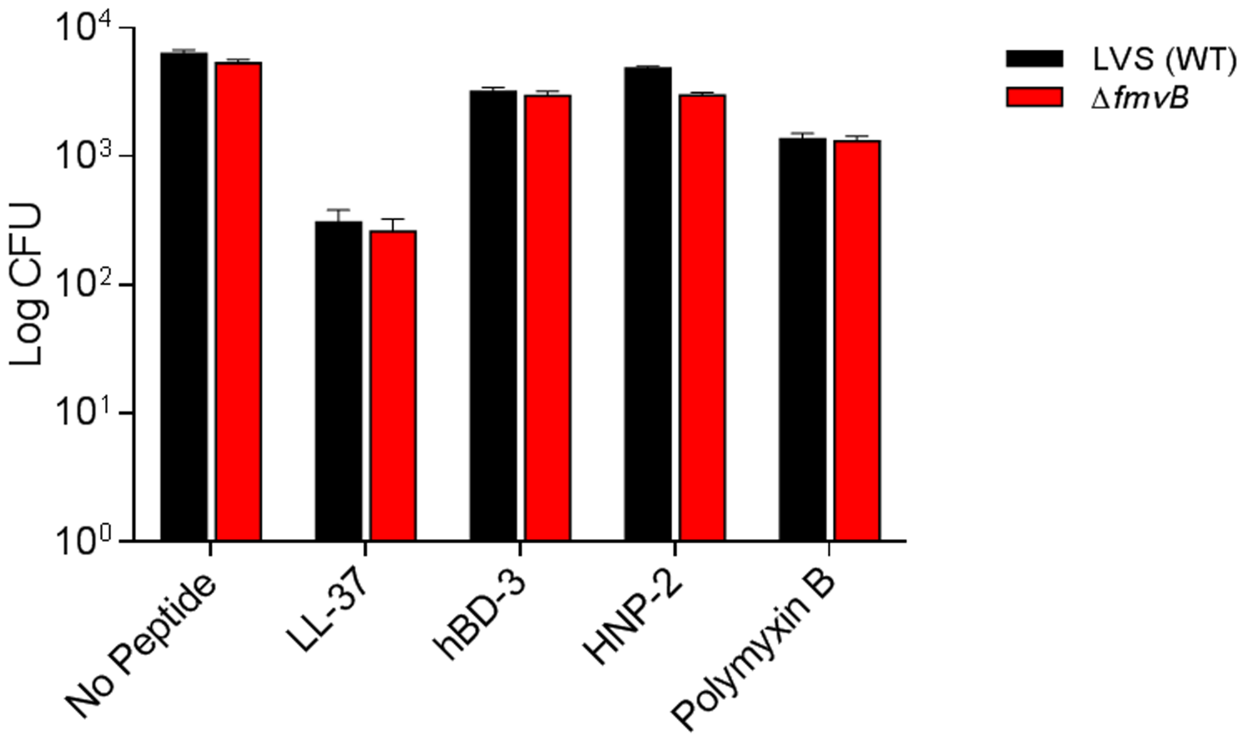

Log CFU
10^4
10^3
10^2
10^1
10^0
LVS (WT)
Δ*fmvB*
No Peptide
LL-37
hBD-3
HNP-2
Polymyxin B

Fig G

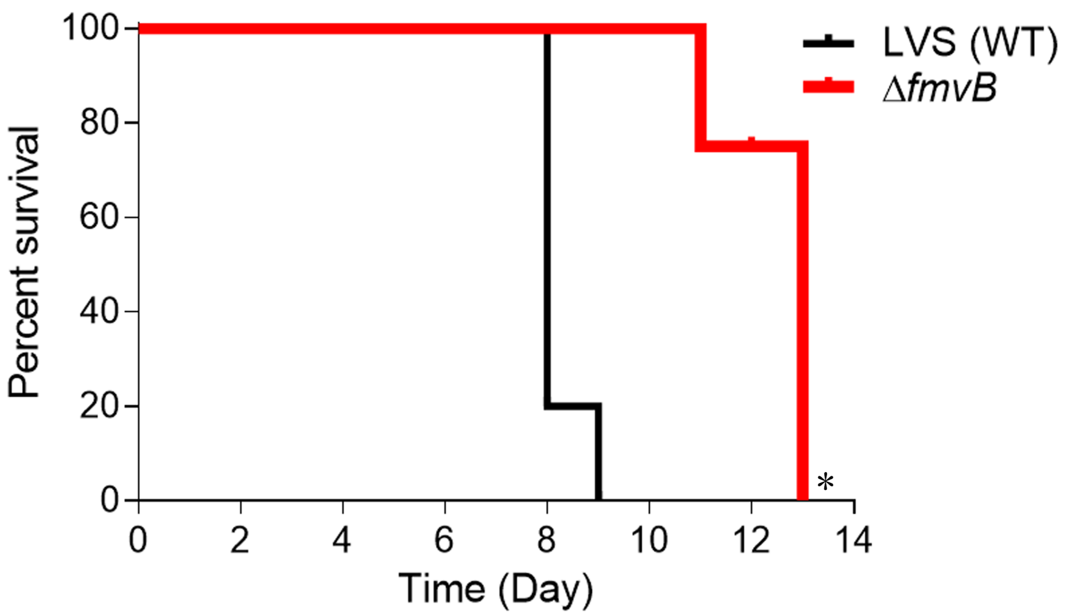

Percent survival
100
80
60
40
20
0
0
2
4
6
8
10
12
14
Time (Day)
LVS (WT)
ΔfmvB
*

Fig H

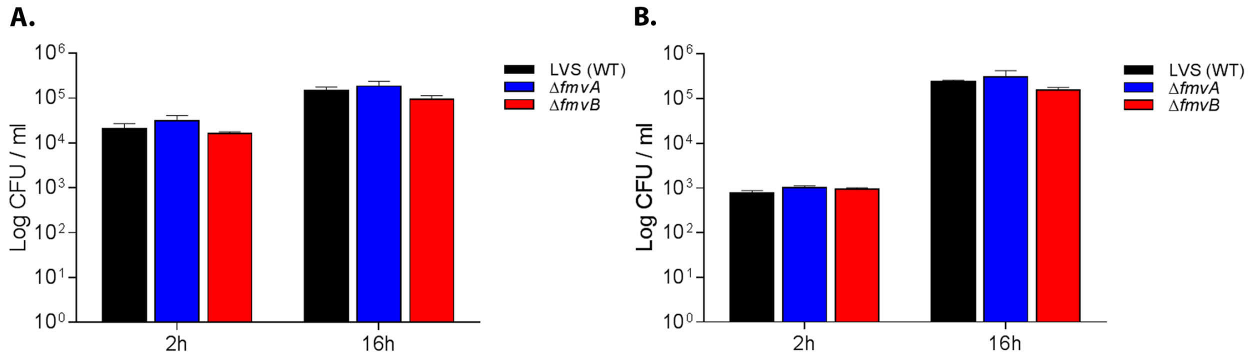

A.
Log CFU / ml
2h
16h
LVS (WT)
ΔfmvA
ΔfmvB
B.
Log CFU / ml
2h
16h
LVS (WT)
ΔfmvA
ΔfmvB

Fig I
